# Targeting fused in sarcoma (FUS): a novel antisense strategy for treating idiopathic pulmonary fibrosis

**DOI:** 10.1038/s41392-026-02585-9

**Published:** 2026-02-26

**Authors:** Bhavika B. Katariya, Shashipavan Chillappagari, Lisa Arnold, Stefan Guenther, Yash Dasadia, Afshin Noori, Ekaterina Krauss, Trushnali Jiyani, Christoph Wrede, Jan Hegermann, Saverio Bellusci, Ludger Fink, Clemens Ruppert, Christian Mühlfeld, Alberto Benazzo, Konrad Hoetzenecker, Clemens Aigner, Andreas Guenther, Poornima Mahavadi

**Affiliations:** 1 Center for Interstitial and Rare Lung Diseases, Department of Internal Medicine, Justus-Liebig University (JLU), Giessen, Germany; 2 Department of Dermatology and Allergology, University Hospital Munich (LMU), München, Germany; 3 Deep Sequencing Platform, Max Planck Institute for Heart and Lung Research, Bad Nauheim, Germany; 4 European IPF Network and European IPF Registry, Giessen, Germany; 5 Hannover Medical School, Institute of Functional and Applied Anatomy, Hannover, Germany; 6 Hannover Medical School, Research Core Unit Electron Microscopy, Hannover, Germany; 7 Biomedical Research in Endstage and Obstructive Lung Disease Hannover (BREATH), Member of the German Center for Lung Research (DZL), Hannover, Germany; 8 Department of Internal Medicine, JLU, Giessen, Germany; 9 Universities of Giessen & Marburg Lung Center (UGMLC), Member of German Center for Lung Research (DZL), Giessen, Germany; 10 Cardio-Pulmonary Institute (CPI), JLU Giessen, Giessen, Germany; 11 Institute of Lung Health (ILH), JLU Giessen, Giessen, Germany; 12 Institute of Pathology, Dermatopathology, Cytology and Molecular Pathology, UEGP, Wetzlar, Germany; 13 Department of Thoracic Surgery, Vienna General Hospital, Vienna, Austria; 14 Department of Thoracic Surgery, Vanderbilt University Medical Center, Nashville, TN USA; 15 Lung Clinic, Agaplesion Evangelisches Krankenhaus Mittelhessen, Giessen, Germany

**Keywords:** Respiratory tract diseases, Translational research

## Abstract

Fused in sarcoma (FUS) is a highly conserved RNA-binding protein with essential roles in RNA processing and genomic stability. While extensively studied in the context of neurodegeneration, its involvement in fibrotic diseases, particularly idiopathic pulmonary fibrosis (IPF), remains largely unexplored. This study investigated the pathological role of FUS in IPF and assessed its viability as a therapeutic target. Specifically, we examine how FUS dysregulation contributes to fibrotic signaling and evaluate whether therapeutic silencing of FUS offers a rational strategy to modulate disease progression. To assess the effects of FUS overexpression and knockdown, functional assays were performed on primary lung fibroblasts derived from healthy donors and IPF patients. Precision-cut lung slices (PCLs) and 3D alveolosphere cultures from IPF patients were treated with a FUS-targeted antisense oligonucleotide (ASO;ION363). FUS-RNA interactions were mapped via CLIP-Seq, and global transcriptional changes following FUS inhibition were analyzed via RNA sequencing. FUS overexpression in healthy fibroblasts promoted proliferation, whereas FUS knockdown attenuated the hyperproliferative phenotype in IPF fibroblasts. IPF cells demonstrated aberrant cytoplasmic mislocalization of FUS. Standard-of-care treatments (pirfenidone, nintedanib) reduced FUS expression in PCLs. CLIP-Seq revealed that FUS binds to a distinct set of profibrotic RNAs in IPF. ION363 treatment downregulated fibrotic gene programs, including those linked to ECM remodeling, TGFβ signaling, and epithelial dysfunction. In contrast, ION363 promoted functional marker expression and improved morphology in patient-derived 3D alveolospheres. We conclude that FUS is a pivotal regulator of fibrotic signaling in IPF and that targeting FUS via ASO represents a promising therapeutic avenue for IPF.

## Introduction

The fused in sarcoma (FUS) protein is a highly conserved RNA-binding protein (RBP) involved in a wide array of cellular functions essential for maintaining cellular homeostasis.^1,2^ FUS functions in RNA transcription, splicing, transport, and translation regulation, and plays a key role in the DNA damage response by supporting genomic stability through DNA repair. Structurally, it contains a prion-like domain, RNA recognition motifs, and a nuclear localization signal, enabling dynamic interactions with nucleic acids and proteins.^3,4^ Under normal conditions, FUS is localized primarily in the nucleus; however, it can translocate to the cytoplasm in response to specific stress signals, demonstrating its functional adaptability.^2,4^ FUS mutations cause cytoplasmic mislocalization and aggregation, contributing to neurodegeneration in patients with amyotrophic lateral sclerosis (ALS) and frontotemporal dementia (FTD) by disrupting RNA metabolism and cellular function.^5,6^ Specifically, mutations in the *FUS* gene account for about 10% of familial ALS^7^, and over 50 variants have been identified thus far.^8^ Most of the variants are in the vicinity of the nuclear localization signal of the *FUS* gene, thereby causing its cytoplasmic mislocalization. Such accumulation in motor neurons recruits the formation of membrane-less compartments called stress granules that serve as temporary reservoirs for FUS-bound RNAs.^9^ However, the failed activation of cellular stress response mechanisms due to the ALS-FUS mutations affects the stress granule dynamics, thereby increasing vulnerability of such cells to further external or harmful stimuli.^9^ In a disease-relevant mouse model of ALS-FUS mutations, a non-allele-specific FUS antisense oligonucleotide (ASO) named ION363 not only silenced the *Fus* gene but also reduced the postnatal levels of FUS protein in the brain and spinal cord, subsequently delaying motor neuron degeneration. Further, it has been reported that, in one ALS patient with a FUSP525L mutation, repeated, intrathecal infusions of ION363 decreased both wild-type and mutant FUS levels in multiple cell types of the central nervous system.^10^ In general, dysregulation of FUS function has also been implicated in age-related cellular dysfunction. Notably, age-related alterations in protein homeostasis may exacerbate FUS mislocalization and aggregation, emphasizing the need for further research into its role in age-associated pathologies.^1,11–13^

Idiopathic pulmonary fibrosis (IPF) is one such age-associated disease with unknown etiology. It represents a major subtype of interstitial lung diseases (ILDs), a heterogeneous group of disorders that significantly contribute to global morbidity and mortality.^14^ IPF is characterized by a continuous loss of lung function and gas exchange properties. Consequently, patients develop persistent dry cough and progressive dyspnea, initially during exertion and later at rest, ultimately culminating in respiratory failure and death, with a median survival of approximately 2–3 years following diagnosis.^15,16^ The characteristic histopathology is usual interstitial pneumonia (UIP), which is characterized by heterogeneity, honeycombing, and fibroblast foci. IPF likely begins with injury to the alveolar epithelium, particularly affecting the alveolar epithelial type II cells (AT2) that are essential for surfactant production and alveolar repair. Epithelial damage and dysfunction lead to the release of profibrotic mediators and the disruption of normal epithelial-mesenchymal signaling, thereby triggering fibroblast activation. The resulting excessive deposition of extracellular matrix (ECM) progressively alters lung structure and mechanics, reinforcing pathogenic feedback loops that sustain fibrotic remodeling to promote disease progression.^14,17^ Currently, pirfenidone and nintedanib are the only antifibrotic therapies approved for the treatment of IPF. Although both agents have been shown to slow the lung function decline, neither halts nor reverses the disease progression.^14–16^ More recently, a third drug, nerandomilast/jascayd, has been approved by the FDA for the treatment of IPF in adults,^18^ but the impact of this drug on long-term survival is awaited. Taken together, IPF continues to be associated with substantial morbidity and mortality, underscoring the urgent need to identify more effective therapeutic strategies that can meaningfully alter the disease course and improve patient outcomes.

Given that dysregulation of RBPs is increasingly recognized as a feature of perturbed tissue homeostasis and in aging, we hypothesized that investigating the role of RBPs, especially FUS, in IPF may provide novel insights into mechanisms linking age-related molecular dysfunction to fibrotic lung remodeling. Elucidating FUS-associated pathways in IPF could therefore uncover previously unrecognized contributors to disease initiation or progression and help identify novel molecular targets relevant to this fatal disorder.

In this study, we systematically examined the role of FUS in IPF and its therapeutic potential. By integrating IPF patient-derived primary lung fibroblasts, precision-cut lung slices (PCLs), and 3D alveolosphere/alveolar organoid culture systems with transcriptomic and RNA-protein interaction analysis, we aimed to address a critical gap in the field regarding the contribution of FUS to fibrotic lung disease. Importantly, this study explored the therapeutic potential of FUS activity using a targeted antisense oligonucleotide approach in patient-derived preclinical models, thereby establishing the relevance of FUS biology in pulmonary fibrosis. Notably, this study is the first to interrogate the function of FUS in IPF and provides a conceptual and experimental foundation for targeting FUS in IPF.

## Results

### Increased cytosolic FUS in IPF fibroblasts

**Fig. 1:**
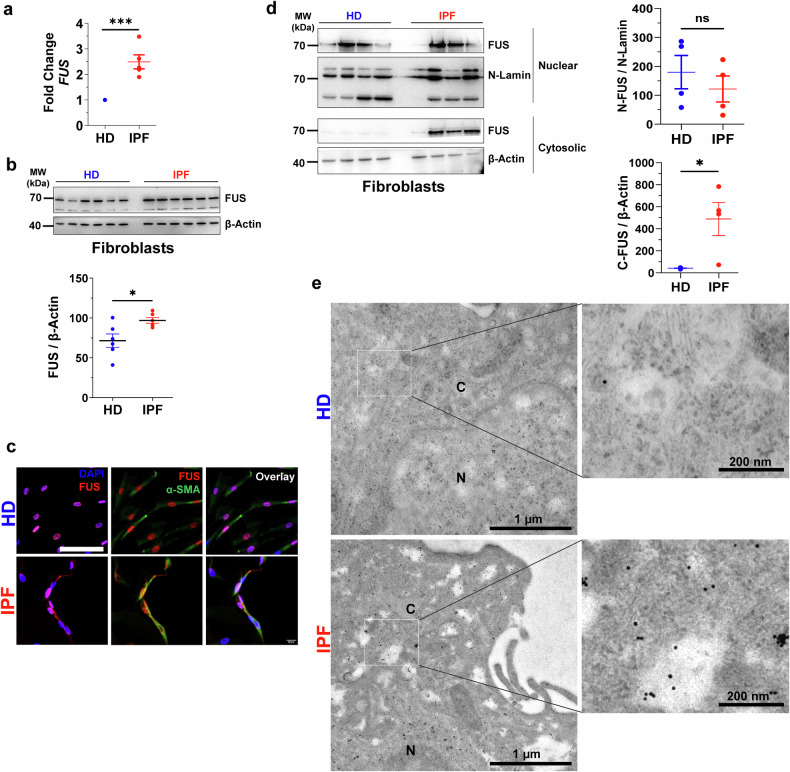
Cytosolic FUS is increased in the interstitial fibroblasts of IPF patients. **a** Analysis of *FUS* mRNA via qRT‒PCR in fibroblasts derived from healthy (HD) or IPF lungs (*n* = 5 HD, *n* = 5 IPF). The values were normalized to those of the housekeeping gene β-actin (*ACTB*). The mean *FUS* mRNA expression in HD fibroblasts was set as one. **b** Immunoblot analysis of FUS or β-actin in total lysates of fibroblasts derived from the lungs of HD or IPF patients. Quantification was performed after normalizing the integrated density values (IDVs) of FUS to those of β-actin. Blots and analyses of fibroblasts from *n* = 6 HDs and *n* = 6 IPF patients. **c** Immunofluorescence analysis of FUS (red) and the fibroblast marker α-smooth muscle actin (α-SMA, green) in cultured fibroblasts from HD and IPF patient lungs. DAPI was used for nuclear staining. Scale bar = 100 µm. **d** Nuclear and cytosolic fractions were enriched from the fibroblasts of HD and IPF lungs, and western blotting was performed for FUS, N-Lamin (nuclear marker), or β-actin (cytosol marker). The right side shows quantification after normalization of the IDV of FUS vs. N-Lamin or FUS vs. β-actin. Fractionation was performed from fibroblasts of *n* = 4 HDs and *n* = 4 IPF. *P*-value summary from (**a**–**d**): *P < 0.05, ***P < 0.001, ns = not significant. **e** Transmission electron microscopy of immunogold-labeled samples. Top: HD cells are labeled predominantly in the nucleus. Bottom: IPF cells show increased labeling in the cytoplasm compared with HD cells. N: Nucleus; C: Cytoplasm

We initially investigated whether FUS is altered in interstitial fibroblasts derived from IPF patients and, if so, whether FUS plays a role in regulating their increased proliferation. For this purpose, we isolated primary interstitial fibroblasts from the explanted lungs of IPF patients or healthy donors (HDs) and performed q‒PCR to analyze FUS gene expression and immunoblotting and immunofluorescence to study FUS protein expression. Interestingly, IPF fibroblasts presented markedly increased FUS expression at both the gene (Fig. 1a) and protein levels (Fig. 1b). Additionally, a significant increase in FUS protein expression was observed in the total lung tissue of IPF patients compared with HD patients (Supplementary Fig. 1a, b). Furthermore, immunofluorescence analysis revealed the nuclear localization of FUS in both HD and IPF cultured fibroblasts. Similarly, FUS was also detected in the cytosol of IPF fibroblasts but not in that of HD fibroblasts (Fig. 1c). To further confirm this, we enriched the nuclear and cytosolic fractions from HD and IPF fibroblasts and probed them with a FUS antibody. This revealed a significant increase in FUS protein in the cytosolic fraction of IPF fibroblasts compared with that in HD fibroblasts. Nuclear FUS remained unaltered (Fig. 1d). To precisely examine FUS localization at the ultrastructural level, we conducted immunogold labeling for FUS in primary HD and IPF interstitial fibroblasts and visualized it via electron microscopy. As shown in Fig. 1e, gold labeling for FUS was visible in the nuclear compartment of both HD and IPF fibroblasts. However, the cytosolic compartment in IPF fibroblasts presented increased FUS labeling, which was not observed in HD fibroblasts. These findings confirm an overall increase in FUS, particularly in the cytosol, in IPF fibroblasts compared with HD fibroblasts. Additionally, other RBPs, namely, TDP43, PABPC1, and MBNL1, were also upregulated in IPF fibroblasts (Supplementary Fig. 1c–h).

### FUS drives the proliferation of IPF fibroblasts

**Fig. 2:**
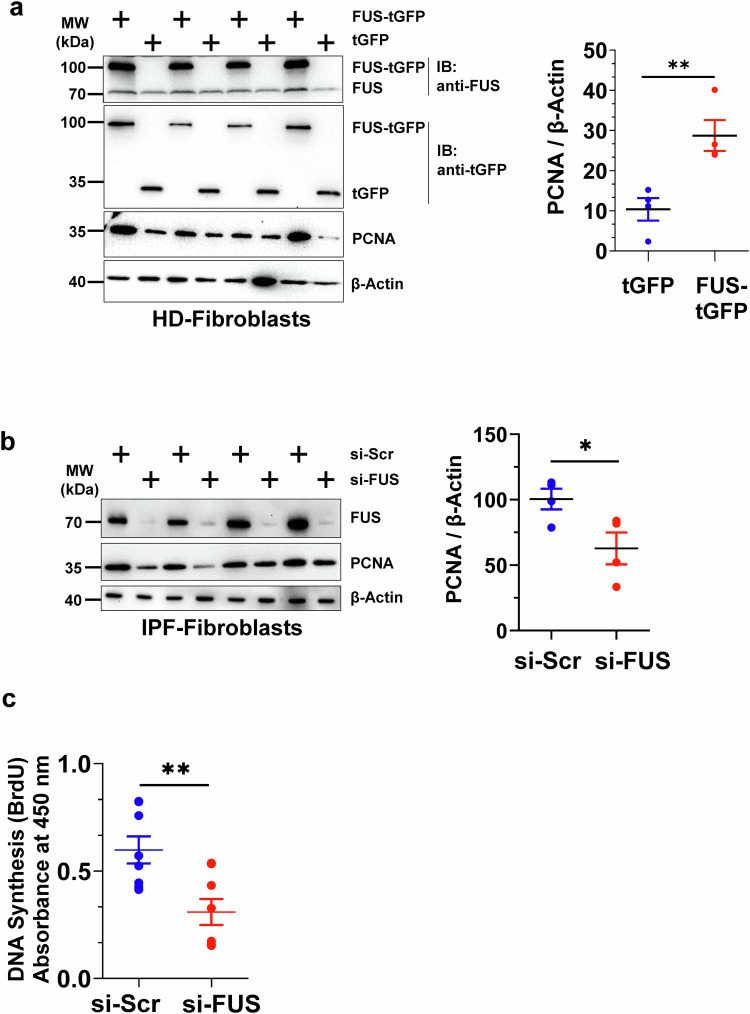
FUS has a proproliferative role in IPF fibroblasts. **a** HD fibroblasts were transfected with plasmids encoding empty turbo GFP (tGFP) or FUS-tGFP for 48 h. Cell lysates were prepared, and western blotting was performed for the indicated proteins. The graph shows the IDV of PCNA normalized to that of β-actin. Blots and analyses were performed with HD fibroblasts (*n* = 4). **b** IPF fibroblasts were transfected with either nontargeting siRNA (si-Scr) or FUS siRNA (si-FUS) for 72 h. Cell lysates were prepared, and western blotting was performed for the indicated proteins. The graph below represents IDV PCNA, normalized to β-actin. Blots and analyses were performed with IPF patient fibroblasts (*n* = 4). **c** Proliferation of IPF fibroblasts (*n* = 8) as assessed by BrdU incorporation. *P*-value summary: *P < 0.05, **P < 0.01

Since excessive fibroblast proliferation is central to IPF, we investigated whether FUS drives this process. In gain-of-function experiments, HD fibroblasts transfected with FUS-turbo-GFP (FUS-tGFP) presented increased proliferating cell nuclear antigen (PCNA) levels, indicating increased proliferation (Fig. 2a). Conversely, FUS knockdown via siRNA in IPF fibroblasts reduced PCNA expression (Fig. 2b) and significantly decreased BrdU incorporation, confirming decreased proliferation (Fig. 2c).

### IPF standard-of-care (SOC) drugs inhibit FUS

Pirfenidone and nintedanib are known to inhibit the proliferation of IPF fibroblasts. To investigate whether these drugs act through the FUS pathway, we treated PCLs (Supplementary Fig. 2a) from IPF patients with vehicle control, pirfenidone, or nintedanib. Notably, both drugs significantly reduced FUS staining in IPF fibroblasts within the IPF PCLs (Supplementary Fig. 2b, c). Additionally, PCLs from control/HD lungs were treated with either a vehicle or a profibrotic cocktail (PFC), which led to a substantial increase in FUS staining in alpha-smooth muscle actin (α-SMA)-positive fibroblasts (Supplementary Fig. 2d) and notable upregulation of *FUS* RNA (Supplementary Fig. 2e). These results suggest that the anti-proliferative effects of standard IPF therapies on fibroblasts may operate, at least partially, through the FUS pathway.

### FUS interacts with profibrotic RNAs in IPF fibroblasts

**Fig. 3:**
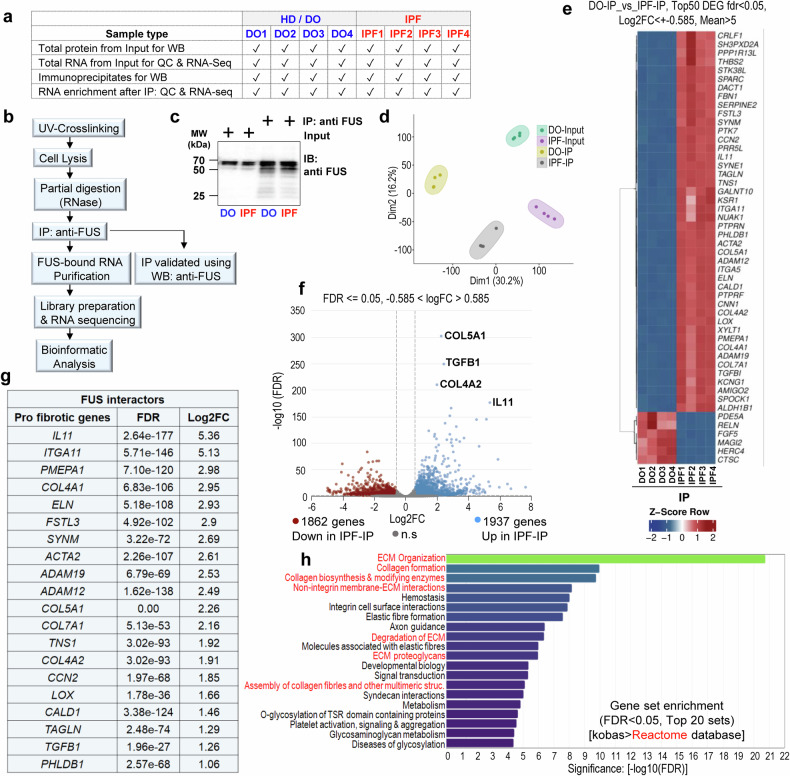
FUS binds to profibrotic RNAs in IPF fibroblasts. **a** Tabular form of samples from HD or IPF fibroblasts used for the experiment involving cross-linking immunoprecipitation and RNA sequencing (CLIP-Seq). **b** Scheme representing the workflow of CLIP-Seq using an anti-FUS antibody for IP. **c** Representative western blot analysis of the CLIP-Seq samples before RNA extraction showing efficient pulldown of the FUS protein. **d** Principal component analysis (PCA) for the multiple groups involved is shown. **e** A Z-score-normalized heatmap of the top 50 DEGs enriched with FUS protein in IPF versus HD fibroblasts is shown. **f** Volcano plot highlighting the top 4 genes, *COL5A1*, *TGFB1*, *COL4A2*, and *IL11*, which are profibrotic genes out of the 1937 upregulated genes/genes bound to FUS in the FUS-IP of IPF fibroblasts. **g** Twenty profibrotic genes that were upregulated/FUS interactors among the top 50 DEGs are listed here, along with the significance (FDR) and log2FC for each gene. **h** Gene set enrichment of the top 50 sets, as analyzed by KOBAS for Reactome, is shown. CLIP-Seq was performed on HD and IPF fibroblasts (*n* = 4 each)

We hypothesized that FUS may promote the proliferation of IPF fibroblasts by binding to RNAs that play a direct or indirect role in proliferative processes and thereby fibrotic processes. To identify the RNAs associated with FUS, we conducted cross-linked immunoprecipitation followed by RNA sequencing (CLIP-Seq). The experimental groups considered for this experiment are shown in Fig. 3a, and the CLIP-Seq experimental setup is given in Fig. 3b for ease of understanding. After confirming successful FUS pulldown via western blotting (Fig. 3c), we performed RNA extraction and sequencing followed by subsequent data analysis. Clusters in the principal component analysis (PCA) highlighted the most notable differences between the groups (Fig. 3d, Supplementary Fig. 3a–c). Significant variation in several genes was observed in IPF-IP (RNAs associated with FUS in IPF fibroblasts) compared with the genes identified in DO-IP (RNAs associated with FUS in HD/DO fibroblasts). Previously identified RNA interactors for FUS were also found in our FUS-RNA interactome. Some examples include *GPR107, SNRNP70, and GAPDH*.^19–21^ Additionally, several novel RNAs were identified, and as expected, those that have been shown to be beneficial to tumor cells for proliferation and migration were identified as FUS interactors in the top 50 DEGs (Fig. 3e). These genes include *NUAK1, PTPRN, ITGA5, CNN1, and PMEPA1*. The top FUS interactors are shown in a volcano plot, namely, the ECM/collagen-associated genes *COL5A1, TGFB1* and *COL4A2* and the profibrotic cytokine *IL11*, which belong to the group of pathologically relevant profibrotic genes (Fig. 3f). Additionally, 20 of the top 50 DEGs were profibrotic genes (Fig. 3g). Interestingly, pathway enrichment analysis of the Reactome database revealed a significant number of genes associated with ECM organization, followed by those associated with collagen formation, biosynthesis and collagen-modifying enzymes (Fig. 3h). These data clearly indicate preferential binding of FUS to profibrotic RNAs in IPF fibroblasts. In addition, the top 50 DEGs from CLIP-Seq for FUS in DO-IP versus DO-Input (Supplementary Fig. 3a, d) and IPF-IP versus IPF-Input (Supplementary Fig. 3b, e) were also analyzed alongside comparisons between the inputs of DO and IPF fibroblasts (Supplementary Fig. 3c, f). These groups served as controls that validated our experimental design and ensured interpretability.

### Downregulation of *FUS* significantly downregulates prosurvival and profibrotic RNAs

**Fig. 4:**
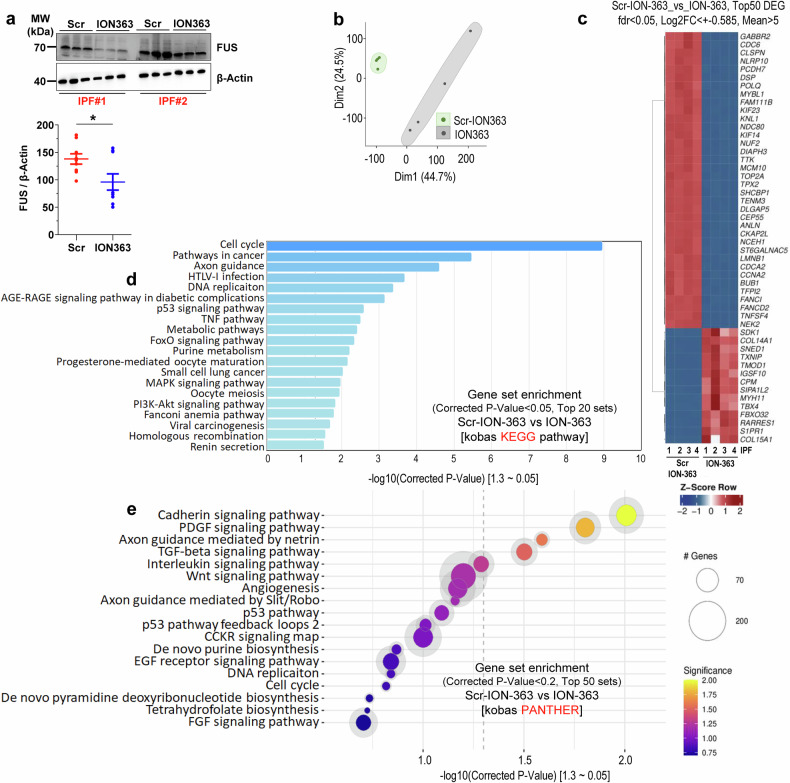
Silencing FUS regulates genes involved in profibrotic signaling in IPF fibroblasts. **a** IPF fibroblasts were either treated with a nontargeting sequence of antisense oligonucleotide (ASO; Scr) or ASO for FUS (ION363; 15 µg) for 72 h. Representative western blots and quantifications from cell lysates of four IPF patient fibroblasts showing a decrease in the protein level of FUS upon ION363 treatment. **b** PCA showing well-separated data sets from these two groups. **c** A Z-score heatmap plot of the top 50 DEGs is shown. **d** Gene enrichment of the top 50 data sets identified via KEGG pathway analysis is shown, with cell cycle genes being the genes most significantly enriched upon ION363 treatment in IPF fibroblasts. **e** Gene enrichment analysis of the top 50 sets via kobas for the PANTHER database, showing genes of the extracellular matrix to be differentially regulated. Blots and analyses were performed on fibroblasts derived from *n* = 4 IPF patients/group

Our data show that FUS promotes fibroblast proliferation in IPF by binding to profibrotic RNAs and that FUS silencing via siRNA reduces cell survival. To validate this, we treated IPF fibroblasts with FUS-targeting antisense oligonucleotide (ASO), ION363, a gapmer ASO designed to lower both wild-type and a neurotoxic FUS variant.^10^ The phosphorothioate and 2′-methoxyethyl modifications of ION363 enhance its stability and efficacy. When a scrambled ASO was used as a control, treatment with ION363 reduced both the RNA and protein expression of FUS in IPF fibroblasts within 48 h (Supplementary Fig. 3a, Fig. 4a). Furthermore, compared with Scr treatment, ION363 treatment resulted in the downregulation of *plasminogen activator inhibitor-1 (PAI-1)*, a factor implicated in the pathogenesis of IPF through its involvement in ECM deposition, as well as *collagen type I alpha 1 (COL1A1)*, a principal structural component of the ECM in IPF fibroblasts (Supplementary Fig. 4b, c). Notably, IPF fibroblasts treated with ION363 presented decreased staining for both nuclear and cytosolic FUS, indicating a decrease in FUS mislocalization in these cells compared with that in Scr-treated IPF cells (Supplementary Fig. 4d). This finding is in line with previous studies that reported decreased levels of pathological FUS within the cytoplasm of ALS patient-derived dermal fibroblasts as well as in the lumbar spinal cord.^10^

Building upon our previous observations shown in Fig. 2, which demonstrated that FUS promotes the proliferation of IPF fibroblasts, we next investigated whether the pharmacological inhibition of FUS by ION363 could mitigate fibroblast migration. To address this, a wound healing assay was performed in IPF fibroblasts treated with either Scr or ION363. As shown in Supplementary Fig. 4e, wound closure was markedly reduced in ION363-treated cells, indicating that ION363 attenuates IPF fibroblast migration, an effect not observed in Scr-treated controls.

Given the apoptosis-resistant phenotype of IPF fibroblasts, we next investigated whether ION363 treatment induces apoptosis in these cells. To this end, immunofluorescence staining for the cleaved form of poly (ADP-ribose) polymerase 1 (PARP1), a well-established marker of apoptosis, was performed. No significant difference in cleaved PARP1 staining was observed between the Scr- and ION363-treated IPF fibroblasts (Supplementary Fig. 4f). Furthermore, as cellular senescence is a well-documented contributor to the aberrant activation of IPF fibroblasts and disease progression, we examined whether ION363 affects fibroblast senescence in culture. Senescence-associated β-galactosidase staining revealed no notable changes in β-galactosidase activity following ION363 treatment compared with that in Scr-treated cells (Supplementary Fig. 4g). These findings indicate that ION363 does not influence apoptotic or senescent pathways in IPF fibroblasts, suggesting that its antifibrotic effects are likely mediated through alternative mechanisms.

We next sought to elucidate the molecular pathways affected by ION363 treatment. Hence, following ASO (Scr/ION363) treatment of IPF fibroblasts, total RNA was extracted, and RNA-seq analysis was conducted after rRNA depletion. PCA revealed a distinct separation between the treatment groups (Fig. 4b). Bioinformatic analysis revealed substantial differences in gene expression profiles between ION363-treated IPF fibroblasts and scrambled controls. Specifically, a heatmap of the top 50 DEGs highlighted these differences (Fig. 4c). Notably, 14 of the top 50 DEGs were upregulated by ION363 treatment, although they did not reveal enrichment of a defined pathway. In-depth analysis revealed a pronounced regulation of genes associated with cell cycle processes, as well as genes linked to cancer-related pathways (KEGG pathway analysis via KOBAS, Fig. 4d), in ION363-treated IPF fibroblasts. Additionally, genes involved in various metabolic pathways were differentially regulated in response to ION363. Further KOBAS analysis in the PANTHER database revealed DEGs associated with the TGF-beta, cadherin, and PDGF signaling pathways (Fig. 4e). Extended pathway analysis via KOBAS for the GO and Reactome databases revealed DEGs associated with multiple pathways, notably those related to cell cycle regulation, in ION363-treated fibroblasts (Supplementary Figs. 5 and 6). Analysis with the KEGG disease database identified DEGs implicated in the pathology of several metabolic and cardiovascular diseases in response to ION363 treatment (Supplementary Fig. 7), indicating that a broad range of substrates are affected by ION363.

**Fig. 5:**
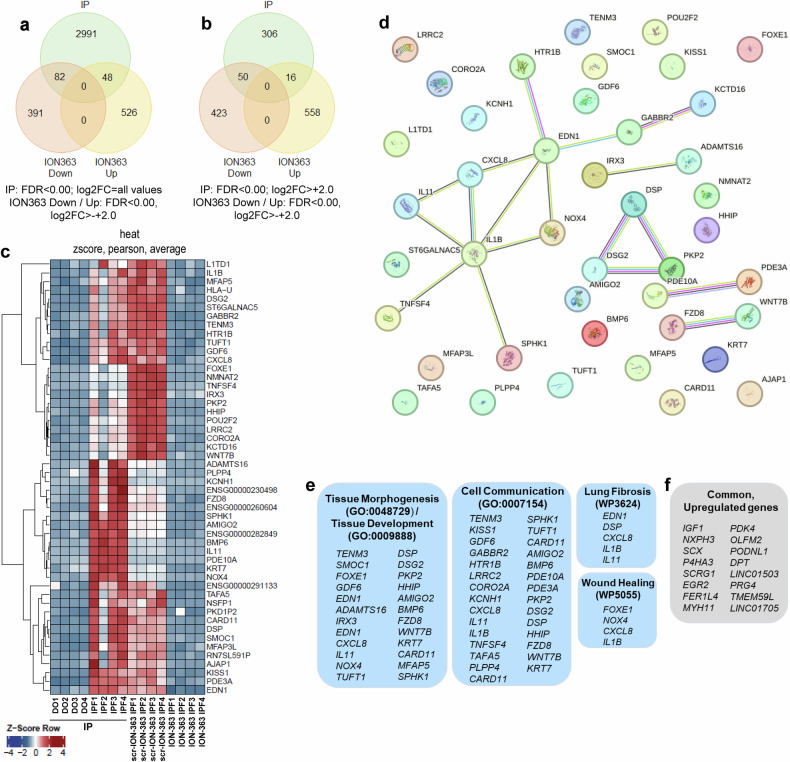
ION363 regulates multiple profibrotic, FUS-interacting genes in IPF fibroblasts. The data set from CLIP-Seq (for FUS, Fig. 1) was compared with the data set obtained from ION363-treated IPF fibroblasts (Fig. 5). **a** Venn diagram of the comparison: With the indicated FDR and log2FC cutoff values, approximately 82 genes that were identified as FUS interactors were downregulated by ION363, and approximately 48 FUS interactor genes were upregulated by ION363. **b** A Venn diagram with a more stringent analysis of our data set is shown, where the stringency was set to FDR < 0 and log2FC > 2.0 for the CLIP-Seq data. In IPF fibroblasts, 50 FUS-interacting genes were downregulated, and approximately 16 were upregulated upon ION363 treatment. **c** A Z-score heatmap plot for the 50 FUS-interacting genes whose expression was downregulated by ION363 is shown. **d** STRING analysis of these 50 genes shows the involvement of these genes in known and unknown predicted interactions. **e** Analysis of these 50 genes revealed that these downregulated genes were involved in tissue development, cell communication, lung fibrosis, and wound healing. **f** The 16 FUS-interacting genes that are upregulated by ION363 treatment are shown

Hence, our next objective was to examine the impact of ION363 treatment on the RNA interactors of FUS, as previously identified in Fig. 3. This analysis was crucial for determining whether ION363 functions as an effective modulator of FUS activity in IPF fibroblasts. To investigate this, we compared two sets of data: data from CLIP-Seq analysis of FUS in IPF fibroblasts (IPF-IP versus DO-IP, shown in Fig. 3) and data from ION363 treatment (ION363 treatment versus scramble control, as shown in Fig. 4). From the latter group, we included both groups: the genes whose expression was upregulated and those whose expression was downregulated. As shown in the Venn diagram in Fig. 5a, 82 FUS-interacting genes were downregulated, and 48 genes were upregulated by ION363. By increasing the stringency of this analysis, where we used a cutoff value of log2FC of 2.0 for the FUS-IP data set, we identified that 50 FUS-RNA interactors were downregulated and that only 16 were upregulated upon ION363 treatment in IPF fibroblasts (Fig. 5b). A Z-score normalized heatmap of 50 genes from combined FUS-IP and ION363 experiments from single HD/Do or IPF is shown in Fig. 5c. We first analyzed the 50 downregulated genes via the STRING database to identify known interactions (Fig. 5d), and these genes could be clustered into four categories. ION363 may most likely act by downregulating these genes: genes that play a role in **1**. Organ development/tissue homeostasis/development (24 genes), **2**. Cell communication (29 genes), **3**. Lung fibrosis (4 genes) and **4**. Wound healing-related genes (4 genes) (Fig. 5e). The 16 genes upregulated by ION363 treatment that were also observed via FUS-IP could not be clustered into defined categories (Fig. 5f), again emphasizing the broad effects of ION363 treatment in IPF fibroblasts.

### FUS-ASO-mediated therapeutic intervention for IPF

**Fig. 6:**
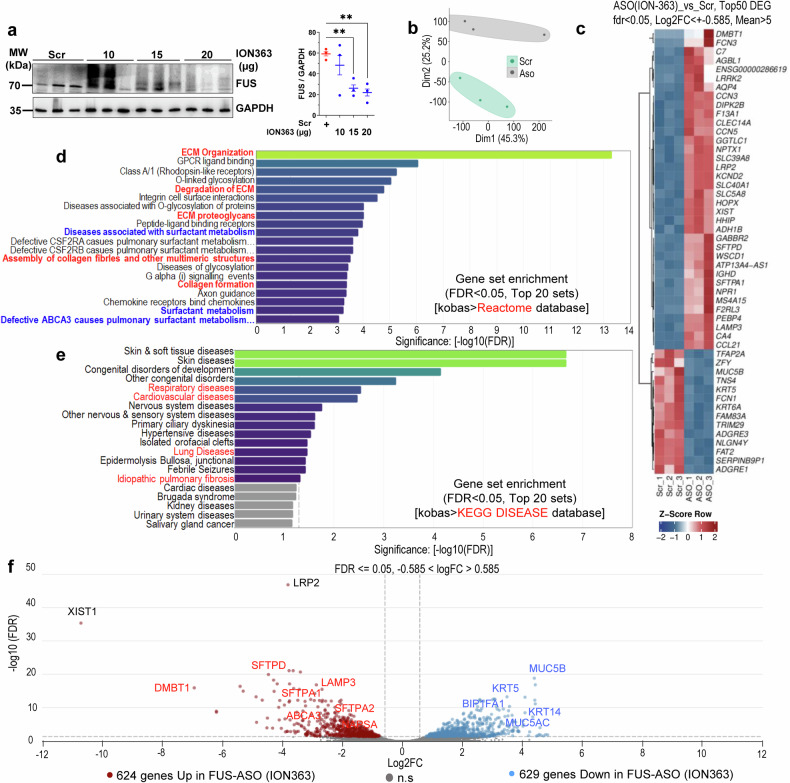
FUS silencing via ION363 treatment ex vivo in IPF PCLs downregulates the expression of multiple profibrotic RNAs. **a** Representative immunoblot images of FUS and GAPDH in IPF PCLs treated with either scramble ASO (Scr) or the indicated doses of ION363 (ASO) for 72 h. Quantification revealed a significant decrease in FUS protein upon ION363 treatment at 15 and 20 µg. **b** PCA plot showing a distinct pattern between Scr and ASO/ION363 treatments. **c** A Z-score heatmap plot of the top 50 DEGs is shown. **d** Gene set enrichment analysis using kobas for the Reactome database, showing differential regulation of genes associated with the extracellular matrix with the highest significance. All the ECM-related groups are highlighted in red. **e** Gene set enrichment via the KEGG disease database showing genes related to skin and soft tissue diseases and genes related to respiratory diseases (red font). **f** Volcano plot of genes whose expression was upregulated (red) or downregulated (blue) in IPF PCLs upon ION363 treatment. Genes that are associated with the greatest log2FC or FDR (*XIST1*, *LRP2*; black font) and genes associated with the alveolar/respiratory epithelium (red font) are also depicted. All analyses were performed on PCLs derived from *n* = 3 IPF patients/group

**Fig. 7:**
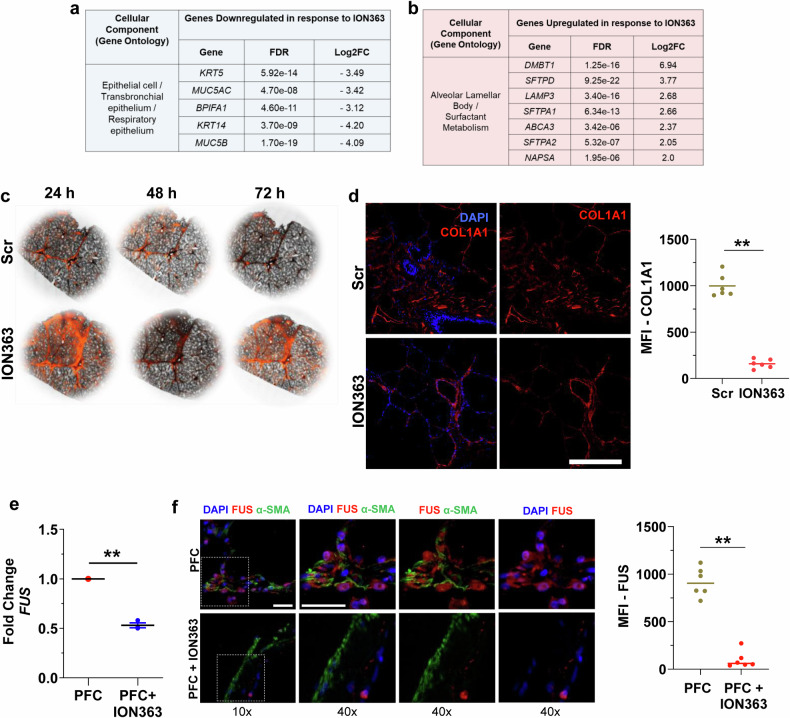
FUS silencing via ION363 treatment ex vivo in IPF PCLs affects multiple pathways. The genes depicted in the volcano plot in Fig. 6f are tabulated in (**a**) (downregulated) and (**b**) (upregulated), with the respective FDR and log2FC values in ION363-treated IPF PCLs. **c** IPF PCLs were treated with scramble ASO (Scr) or FUS-ASO (ION363) followed by LysoTracker Red staining on PCLs, and live imaging of the treated PCLs was performed at the indicated times. The brightfield images depicted here are in combination with the red fluorescence signal of LysoTracker Red. Pictures and analysis of *n* = 1 IPF PCLs are shown. **d** Representative images showing immunostaining for COL1A1 (red) in IPF PCLs treated with Scr or ION363. DAPI was used to stain the nuclei. Scale bar = 300 µm. The graph on the right side represents the COL1A1 mean fluorescence intensity (MFI) in the indicated groups. **e**, **f** HD PCLs were treated with a profibrotic cocktail (PFC) or PFC in combination with ION363, as shown here by the analysis of *FUS* mRNA via qRT‒PCR. **e** Immunofluorescence staining for FUS (red) or α-SMA (green). Nuclei were stained with DAPI. Scale bar for: 10× images = 20 µm, 40× images = 50 µm. **f**. The quantification of the FUS MFI is shown, with pictures and analysis from *n* = 2 HD PCLS. *P*-value: **P < 0.01

Our study investigated the role of FUS and the effects of the therapeutic agent ION363 on FUS expression and other profibrotic mediators, focusing predominantly on fibroblasts derived from IPF patients. Fibroblasts are known to be critical drivers of fibrosis progression; however, to robustly evaluate the therapeutic potential of ION363 for IPF, a more sophisticated model is necessary. Thus, we employed PCL technology. We treated PCLs derived from IPF patients with either Scr-ASO or ION363 in a dose-dependent manner (10, 15, or 20 µg). Significant downregulation of FUS protein expression was observed at 15 µg and 20 µg of ION363 (Fig. 6a, Supplementary Fig. 8). Additionally, 15 µg was identified as the minimum effective dose required to significantly reduce FUS protein levels. Therefore, we selected 15 µg as the dose for subsequent experiments and treated PCLs derived from the explanted lungs of three IPF patients with either Scr-ASO or ION363 at this concentration, and total RNA was extracted as described in the “Methods” section. RNA sequencing was subsequently performed after rRNA depletion. Our data revealed several DEGs in response to ION363 treatment (Fig. 6b, c), and gene enrichment analysis via the KOBAS for the Reactome database revealed significant regulation of the DEGs related to ECM organization (Fig. 6d). Furthermore, KEGG disease database analysis revealed DEGs in the categories of respiratory & cardiovascular diseases as well as IPF (Fig. 6e). We next performed an in-depth analysis of the top 50 DEGs (Fig. 6f) and identified the downregulation of five genes belonging to the GO: epithelial/transbronchial/respiratory epithelium in response to ION363 treatment (Fig. 7a). These genes are *KRT14, MUC5B, KRT5, MUC5AC* and *BPIFA1*. Interestingly, several genes associated with the alveolar epithelial lamellar body and surfactant metabolism were significantly upregulated. These genes included *DMBT1, SFTPD, LAMP3, SFTPA1/A2, ABCA3,* and *NAPSA* (Fig. 7b). Further gene set enrichment of the DEGs via KOBAS for the PANTHER and NHGRI GWASs revealed significant differential regulation of genes belonging to the coagulation cascade and pulmonary function, respectively, again emphasizing the broad potential of ION363 (Supplementary Figs. 9 and S10). Additionally, as we observed an upregulation of epithelial compartmental genes, especially those in the alveolar epithelium, we stained IPF PCLs from one patient with a lysosomal dye, LysoTracker (red), upon Scr-ASO or ION363 treatment. In addition to the upregulation of alveolar epithelial-specific genes, we observed intense staining for LysoTracker in ION363-treated IPF PCLs compared with that in Scr-ASO-treated IPF PCLs (Fig. 7c). In parallel, we performed immunofluorescence staining for COL1A1, a fibrillary collagen associated with fibrotic tissues and an accepted marker of the ECM component that is upregulated in the lung tissues of IPF patients. Compared with those treated with Scr-ASO, IPF PCLs treated with ION363 presented a marked decrease in COL1A1 staining (Fig. 7d). In alignment with the RNA-seq data, we also observed that surfactant protein A (SP-A), an immune-related surfactant, was increased in HTII-280-positive AT2 cells in IPF PCLs treated with ION363 (Supplementary Fig. 8b). Next, we treated HD PCLs with either the PFC alone or a combination of the PFC and ION363 to assess whether ION363 could counteract PFC-induced FUS expression. The results indicated that ION363 effectively downregulated PFC-induced *FUS* expression (Fig. 7e). Consistently, we observed a marked reduction in FUS staining in PFC + ION363-treated HD PCLs compared with those treated with PFC alone (Fig. 7f), suggesting the potential for ION363 to modulate FUS levels.

### FUS silencing enhances alveolar regeneration and epithelial differentiation in IPF-derived alveolospheres

**Fig. 8:**
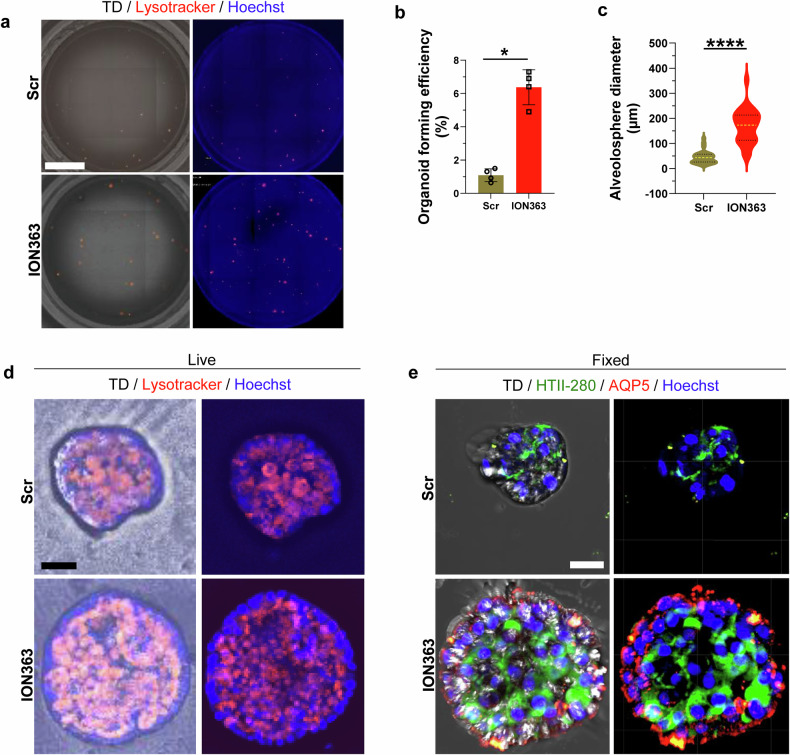
FUS silencing enhances alveolar regeneration and epithelial differentiation in IPF-derived alveolospheres. **a** Alveolospheres treated with Scr-ASO (Scr) or ION363 were cultured until day 21 and stained with LysoTracker Red and Hoechst (nuclei), followed by live imaging at 4× in an EVOS M7000. Stitched images with or without TD (transmitted light/brightfield) were saved via Celleste 6.0. Scale bar = 2 mm. **b** The number of alveolospheres formed per plated cell number in each treatment was counted and quantified, or **c** each alveolosphere was measured via Celleste 6.0 software. *P*-value summary: *P < 0.05, ***P < 0.001. **d** Representative images of one 3D live alveolosphere stained with LysoTracker/Hoechst and imaged via an EVOS M7000 at 20×. **e** Representative imag**e**s of one fixed alveolosphere from each treatment group that was stained with HTII-280 (green), AQP5 (red), or Hoechst (blue) and imaged via an Olympus FV3000 microscope. Scale bar = 20 µm. Representative images and analyses of alveolospheres from *n* = 3 IPF patients are shown

Our previous studies focused primarily on IPF fibroblasts and PCLs. On the basis of the data obtained from our PCLs analysis, especially with the observation that ION363 treatment affects the expression of alveolar epithelial genes, we hypothesized that ION363 may also exert antifibrotic effects on AT2 cells of IPF patients. First, to understand whether FUS is expressed by AT2 cells in HD or IPF lungs, we performed immunocytochemistry on paraffin sections for FUS and the AT2 marker pro SP-C. As indicated in Supplementary Fig. 11a, not only fibroblasts but also AT2 cells presented enhanced staining for both nuclear and cytosolic FUS in IPF AT2 cells. We next isolated AT2 cells from IPF or HD lungs, cultured them, and treated them with Scr or ION363. Interestingly, we observed decreased FUS staining in AT2 cells treated with ION363 (Supplementary Fig. 11b). To further investigate the epithelial-specific impact of FUS silencing, we extended our findings from our bulk RNA-seq analysis of ION363-treated IPF PCLs, which revealed robust transcriptional activation of alveolar epithelial gene programs. Therefore, we sought to functionally assess the regenerative potential of ION363 by employing a 3D alveolosphere model derived from primary AT2 epithelial cells isolated from the lungs of patients with IPF. We generated alveolospheres from AT2 cells of IPF patients without feeder cells (fibroblasts) and treated them with Scr or ION363. However, as shown in Supplementary Fig. 11c, cultures of the alveolosphere alone struggled to survive under Scr-treated conditions. Under conditions of ION363 treatment, several alveolospheres increased in size and number. However, such alveolospheres are difficult to culture and do not survive beyond day 14. We performed live imaging of alveolospheres on day 14 by staining them with LysoTracker Red and nuclear staining with Hoechst, which revealed larger alveolospheres following ION363 treatment (Supplementary Fig. 11d). Next, we generated alveolospheres from IPF AT2 cells (purity of the isolated AT2 cells is shown in Fig. S12) under defined conditions alongside feeder cells (fibroblasts) and treated these alveolospheres with either Scr-ASO or ION363 every third day for a 21-day period. To evaluate cellular viability and morphology, live alveolospheres were stained with LysoTracker and Hoechst, while parallel cultures were fixed with paraformaldehyde for whole-mount immunofluorescence. Strikingly, live imaging revealed increased LysoTracker uptake in ION363-treated alveolospheres, indicating increased lysosomal activity, a hallmark of epithelial renewal (Fig. 8a). Compared with those of the Scr-ASO controls, the quantitative analyses revealed a significantly greater frequency of alveolosphere formation and a marked increase in alveolosphere size following ION363 treatment (Fig. 8b–d, Supplementary Videos V1–V4). Furthermore, whole-mount staining for the AT2 marker HTII-280 and the AT1 marker Aquaporin 5 (AQP5) revealed substantial upregulation of AQP5 expression in ION363-treated cultures, which was consistent with enhanced differentiation toward the AT1 lineage (Fig. 8e). Collectively, these data provide compelling evidence that FUS silencing via ION363 not only restores epithelial gene expression profiles but also functionally enhances epithelial integrity and regenerative capacity in IPF-derived alveolospheres, underscoring its therapeutic potential in promoting alveolar repair.

## Discussion

**Fig. 9:**
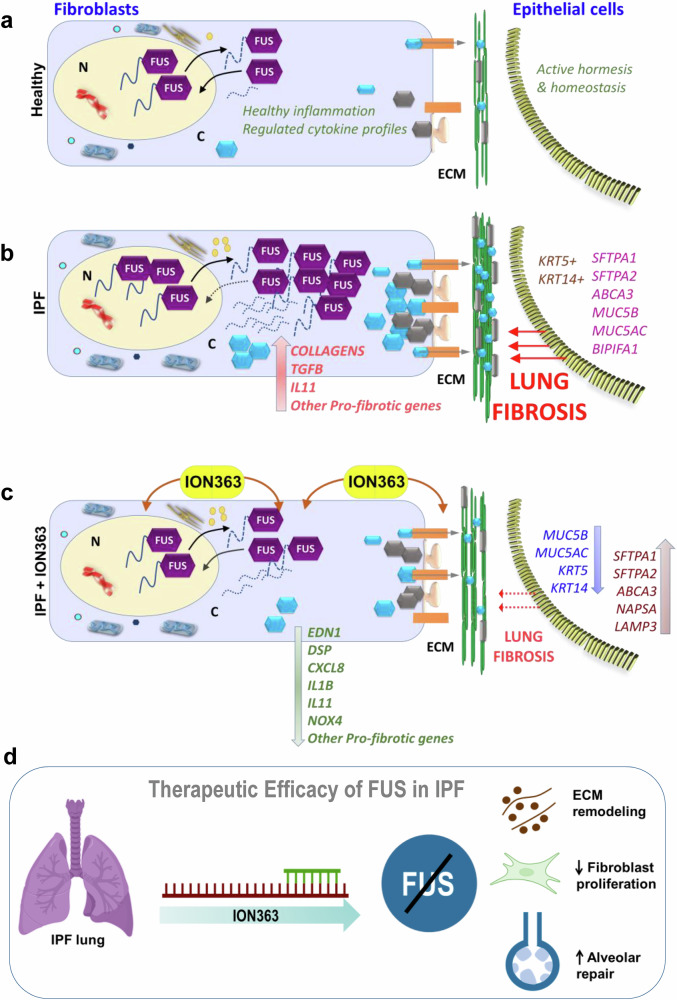
Antisense oligonucleotide silencing of FUS is a novel therapeutic intervention for IPF. Schematic representation of this study showing two cell types, fibroblasts and epithelial cells, under three different conditions: **a** healthy, **b** IPF, and **c** IPF PCLs/cells treated with FUS-ASO or ION363. As depicted, normal levels of FUS protein in the nucleus and healthy nucleus-cytoplasmic shuttling of FUS (solid arrows), accompanied by healthy inflammation, regulated cytokine profiles and active epithelial cell homeostasis, are natural phenomena in healthy cells (**a**). Under IPF conditions, FUS accumulates in the nucleus and more so in the cytoplasm along with its profibrotic client RNAs, which may alter their metabolism. This results in the upregulation of profibrotic gene signatures, namely, *collagens, TGFB, IL11* and other genes of the ECM in fibroblasts. On the other hand, the already injured epithelium (respiratory/alveolar) shows activation of cytokeratins (*KRT5 & 14*) and aberrant surfactant homeostasis (**b**). In response to ION363 treatment, the protein levels of FUS are decreased in fibroblasts, leading to a downregulation of its profibrotic, client RNAs, as depicted. In epithelial cells, *SFTPA1/2, ABCA3, NAPSA,* and *LAMP3 are upregulated, whereas MUC5B/5AC, KRT5,* and *KRT14* are downregulated, indicating decreased profibrotic signaling (**c**). The fibroblast- and epithelial-specific genes among the top 50 DEGs from our RNA-seq analysis are depicted here for a simplified understanding. **d** A consolidated summary: Silencing of FUS in IPF lungs via an antisense oligonucleotide, ION373, restores homeostasis by suppressing fibrotic transcriptional programs (by remodeling the ECM and reducing fibroblast proliferation) and promoting repair processes (alveolar repair and regeneration), supporting its candidacy as a novel therapeutic target in IPF

This study identified a novel role for the RNA-binding protein FUS in IPF pathogenesis. Gain- and loss-of-function experiments in primary fibroblasts revealed that FUS promotes their proliferation. CLIP-seq confirmed that FUS binds to profibrotic RNAs and that treatment with the antisense oligonucleotide ION363 effectively reduced the expression of these targets. RNA-seq of ION363-treated IPF PCLs revealed the suppression of fibrotic gene signatures and the restoration of epithelial-specific genes, leading to alveolar regeneration. This is the first study to link FUS to fibroblast activation, epithelial repair, and AT2 regeneration, positioning it as a promising therapeutic target in IPF (Fig. 9a–d).

Extensive research has established FUS as a critical RNA-binding protein involved in the pathogenesis of ALS^7,22^ and several types of sarcomas.^4,23,24^ FUS has also been strongly linked to aging, with evidence showing that liquid‒liquid phase separation of the FUS protein leads to a transition from liquid droplets to aggregated states over time, a process exacerbated by patient-derived mutations.^25^ More recently, FUS has been identified as a key regulator of hematopoietic stem cell aging.^12^ Consistent with prior findings, our study revealed that IPF, an age-related disease, involves pathological FUS activity. We observed cytoplasmic FUS aggregation in IPF fibroblasts, and its knockdown impaired their proliferation and migration. These results align with earlier studies linking the loss of FUS to disrupted cell proliferation and key pathways such as cell cycle control, cytoskeletal dynamics, and energy metabolism.^26^

Our study is the first to report FUS binding to a subset of profibrotic RNAs. CLIP-Seq analysis revealed that 20 of the top 50 FUS-interacting DEGs aligned with known profibrotic signatures, including key transcripts such as *TGFβ, COL5A1, COL4A2*, and *IL11*, in IPF fibroblasts. We also confirmed FUS associations with previously known RNAs (*Gabra1, Nrxn3, TAF15, and Nvl*^21,27,28^) in healthy fibroblasts, although some expected targets were absent, likely due to the tissue- and cell-specific roles of FUS. FUS exerts multifaceted control over RNA metabolism, influencing processes including splicing, stability, localization, and translation in a context-dependent manner. Under cellular stress, FUS can undergo liquid‒liquid phase separation to form stress granules, membrane-less compartments that transiently sequester untranslated mRNAs to protect them from degradation.^29,30^ Persistent stress granule formation and cytoplasmic aggregation of mutant FUS are well-documented pathogenic features of FUS-associated ALS, indicating a gain-of-toxicity function.^9^ However, in the context of IPF fibroblasts, where FUS is largely expressed in its wild-type form, our findings that FUS binds multiple profibrotic RNAs likely reflect its physiological, non–stress-related functions. Thus, FUS may modulate the stability, translation, or localization of these transcripts rather than mediating their sequestration. Collectively, these observations suggest that FUS contributes to the profibrotic phenotype of IPF fibroblasts through regulatory effects on target RNA networks, although the precise mechanisms of this regulation remain to be elucidated.

Our study is supported by the use of a translationally relevant antisense oligonucleotide therapy targeting FUS, specifically ION363. This ASO has been demonstrated to bind FUS mRNA, inhibiting its translation and thereby reducing FUS protein levels. Schneider and colleagues^10^ provided compelling evidence that ION363 can effectively lower FUS expression in preclinical mouse models of ALS-FUS and in a human patient harboring an ALS-FUS mutation. Currently, a phase 3 clinical trial is underway to assess the efficacy of ION363 in improving clinical function and survival in individuals carrying FUS-ALS mutations (NCT04768972). In ALS, in addition to the nervous system, several peripheral tissues exhibit significant fibrosis and inflammation, contributing to tissue degeneration.^31^ Recent studies have further demonstrated the cytoplasmic retention of FUS in animal models of kidney and liver fibrosis.^13^ Our study advances FUS biology by identifying novel FUS-interacting RNAs in healthy and fibrotic lung tissue and demonstrating that ION363 reduces profibrotic signatures in IPF patient-derived PCLs. ION363 also modulates key respiratory and alveolar epithelial genes, including *MUC5B, MUC5AC, KRT5, KRT14, SFTPA1/2, ABCA3, NAPSA, and LAMP3*, with MUC5B promoter polymorphisms being the most common genetic risk factor for IPF.^32^ Additionally, recent research has identified two tightly linked low-frequency single-nucleotide variants in MUC5AC as further risk factors for this disease.^33^ Next, both *KRT5* and *KRT14* expression were reported, especially in the honeycombing regions of IPF lungs. Single-cell RNA-seq analysis of IPF lungs suggested that *KRT5*^*+*^ basal cell expansion in the distal lung epithelium is accompanied by the progressive loss of alveolar epithelial cell loss and tissue remodeling^34^ and that genes of the ECM influence the migration of these cells.^35^ In addition to KRT5, KRT14 has been shown to be elevated in the alveolar regions of IPF lungs.^36^ Notably, ION363 treatment significantly downregulated these genes in IPF PCLs, indicating its relatively broad mode of action. Variants in SFTPA1/2 and ABCA3 are linked to pulmonary fibrosis and interstitial lung disease,^37^ whereas LAMP3 mutations affect lamellar body function in pediatric ILD patients.^38,39^ LAMP3 is another lamellar body-associated protein, and biallelic *LAMP3* variants have been recently identified in children with interstitial lung disease.^40^ ION363 upregulated NAPSA in IPF PCLs, which aligns with previous reports of Napsin A loss in AT2 cells, as loss or inhibition of Napsin A contributes to AT2 injury and lung fibrosis.^41,42^ These data suggest that pharmacological inhibition of FUS increases IPF- AT2 regeneration, indicating its potential as an antifibrotic therapy.

Our study relies heavily on human-derived translational models to elucidate the molecular mechanisms of FUS function in IPF. We believe that human tissues and 3D ex vivo systems, as shown in our study, are translationally relevant platforms for assessing the antifibrotic mechanisms of FUS inhibition. However, some limitations merit mention. Extending the antifibrotic mechanisms of FUS inhibition in in vivo models of lung fibrosis may help to fully capture physiological complexity. Next, the proteomic analysis shown in our study captures selected pathways/proteins rather than a global analysis. Recognizing these boundaries while interpreting the results presented in this study will place this work in context and open new directions that may further enhance our understanding.

A key finding of our study is that ION363 not only reduces FUS protein levels but also impacts IPF AT2 cell regeneration and positively influences multiple antifibrotic pathways, including the ECM, the cell cycle, coagulation, and epithelial regeneration. This broad activity resembles that of the approved IPF drugs pirfenidone and nintedanib, which target the heterogeneous nature of this disease. The multifactorial pathology of IPF calls for therapies with complementary or synergistic mechanisms.^43^ The wide-ranging effects of ION363 make it a promising therapeutic candidate, potentially enhancing current treatments by addressing diverse disease drivers and improving patient outcomes.

## Materials and methods

The human lungs used in this study were explanted from patients with sporadic IPF as well as from nondiseased control subjects, including healthy donors and organ donors (HDs), via the Department of Thoracic Surgery in Vienna. Information on the patients and samples used across various experiments can be found in Supplementary Table 1. All IPF diagnoses followed the consensus criteria of the American Thoracic Society/European Respiratory Society. Ethical approval for the study protocol was granted by the Ethics Committee of the Justus-Liebig University School of Medicine (111/08 and 58/15). The patient data and biomaterials (Supplementary Table 1) used were supplied by the UGMLC Giessen Biobank and the European IPF Registry/Biobank.

### Primary human lung fibroblasts

Primary human lung fibroblasts were isolated from explanted IPF and control lungs following an outgrowth technique as described before^44–46^, and all experiments were undertaken using cells from passage # 2 or 3. Cells were grown in DMEMF-12 medium (with or without phenol red) supplemented with 10% FCS, 1% Pencillin / streptomycin, 1% non-essential amino acids, and 1% L-Glutamine. Nuclear and cytosolic fractions were enriched using a nuclear/cytosol fractionation kit (Abcam). Colorimetric bromodeoxyuridine (BrdU) assay (Sigma) was performed as described before 4 by allowing cells to grow in a 96-well plate. Primary interstitial fibroblasts from HD lungs were transfected with empty turbo GFP (tGFP, Origene) or FUS-tGFP (Origene) using lipid based method (lipofectamine, Thermo Fisher Scientific). Primary interstitial fibroblasts from IPF lungs were transfected with nontargeting / scramble siRNA (Santa Cruz) or with FUS siRNA (Santa Cruz) using Lipofectamine.

### Precision-cut lung slices (PCLs)

1.5–3% low melting agarose (maintained at 37 °C) was filled in each segment of the explanted human IPF/HD lung and was allowed to cool on ice for 30 min for the agarose to solidify. A vibrating blade microtome (Thermo Fisher Scientific) was used to section blocks of tissue filled with agarose. About 500-µm-thick sections were made and cultured in RPMI medium without phenol red, supplemented with 2% FCS, 1% penicillin/streptomycin, and 1% L-Glutamine. PCLs were left for 24–48 h in a cell culture incubator. PCLs were cultured in 6-well plates by pooling about 3–5 slices in one well, depending on the size of the PCLs used. *n* of 3 IPF patient lungs were used for the experiment,s undertaking PCLs samples each for Scr-ASO (Qiagen) or ION363 treatment (MedChem Express) in the indicated concentrations or with Pirfenidone (2.7 mM) or Nintedanib (300 µM) as described before.^46^ HD PCLs were treated with pro-fibrotic cocktail (PFC) comprising TNFα (10 ng/mL; R&D Systems), TGFβ (5 ng/mL; R&D Systems), LPA (5 µM; Cayman Chemical), and PDGF (10 ng/mL; R&D Systems). Before treatments, PCLs were washed with PBS. Following treatments, PCLs were fixed in formalin and embedded in paraffin or were shock-frozen and processed for RNA extraction as described above.

### Cross-linking immunoprecipitation & RNA sequencing (CLIP-Seq)

CLIP-Seq for FUS was performed following protocols that were described before.^47^ Briefly, HD and IPF fibroblasts were irradiated with UV light (100 mJ/cm^2^, 254 nm in Startalinker) to achieve irreversible protein-RNA interactions. Cells were lysed using ice-cold RNA lysis buffer (with SUPERase & protease inhibitors), scraped, and collected. Collected samples were pipetted thoroughly, followed by incubation on ice. About 20 µL of this sample was then aliquoted to be used as input for the western blot. Samples were then incubated with anti-FUS antibody overnight, rotating at 4 °C. The next day, protein A/G beads (Abcam) were washed, resuspended in RNA lysis buffer, and added to the sample (containing FUS antibody). Following 2 h of incubation at 4 °C, samples were washed thrice with DPBS, and 10 µL of beads were collected for RNA-IP control for western blot analysis. Next, proteinase K digestion was performed for 10 min at 37 °C, 1100 rpm and samples were immediately transferred onto the ice, and 0.4 mM PMSF was added to deactivate proteinase K. This was followed by RNA extraction using Qiazol RNA isolation kit (Qiagen) as per the manufacturer’s instructions.

### Statistics

Data are expressed as means ± SD. At least three independent experiments were performed. At least 3 PCLs from each IPF patient were pooled, and PCLs from three IPF patients were used for the treatment with Scr-ASO/ION363. The data did not follow normal distribution, hence statistical significance was assessed using non-parametric tests: the Mann–Whitney U test for comparing two groups. *P*-value summary: *P ≤ 0.5, **P ≤ 0.01, ***P ≤ 0.001.

Further details about alveolosphere generation and culture, electron microscopy, qRT-PCR, primers used (Supplementary Table 2), western blot and immunofluorescence, antibodies used in this study (Supplementary Table 3), and RNA sequencing are given in the online supplement. Microsoft PowerPoint and Biorender were used to generate schematic illustrations.

## Supplementary information

Video 1

Video 2

Video 3

Video 4

Videos1 - 4

Supplementary Methods, Tables, references, and figure legends-Clean version

## Data Availability

Additional data are available as online supplementary figures. The data associated with this study have been deposited at the GEO repository under the accession number GSE310479.
