# Supplementary material for: Targeting fused in sarcoma (FUS): a novel antisense strategy for treating idiopathic pulmonary fibrosis
Source: Signal Transduct Target Ther. 2026 Feb 26;11:70. doi: 10.1038/s41392-026-02585-9 (PMC12936215; doi:10.1038/s41392-026-02585-9)
Supplement: Supplementary file 5 — Videos1 - 4 [file 41392_2026_2585_MOESM5_ESM.pptx]

## Slide 1
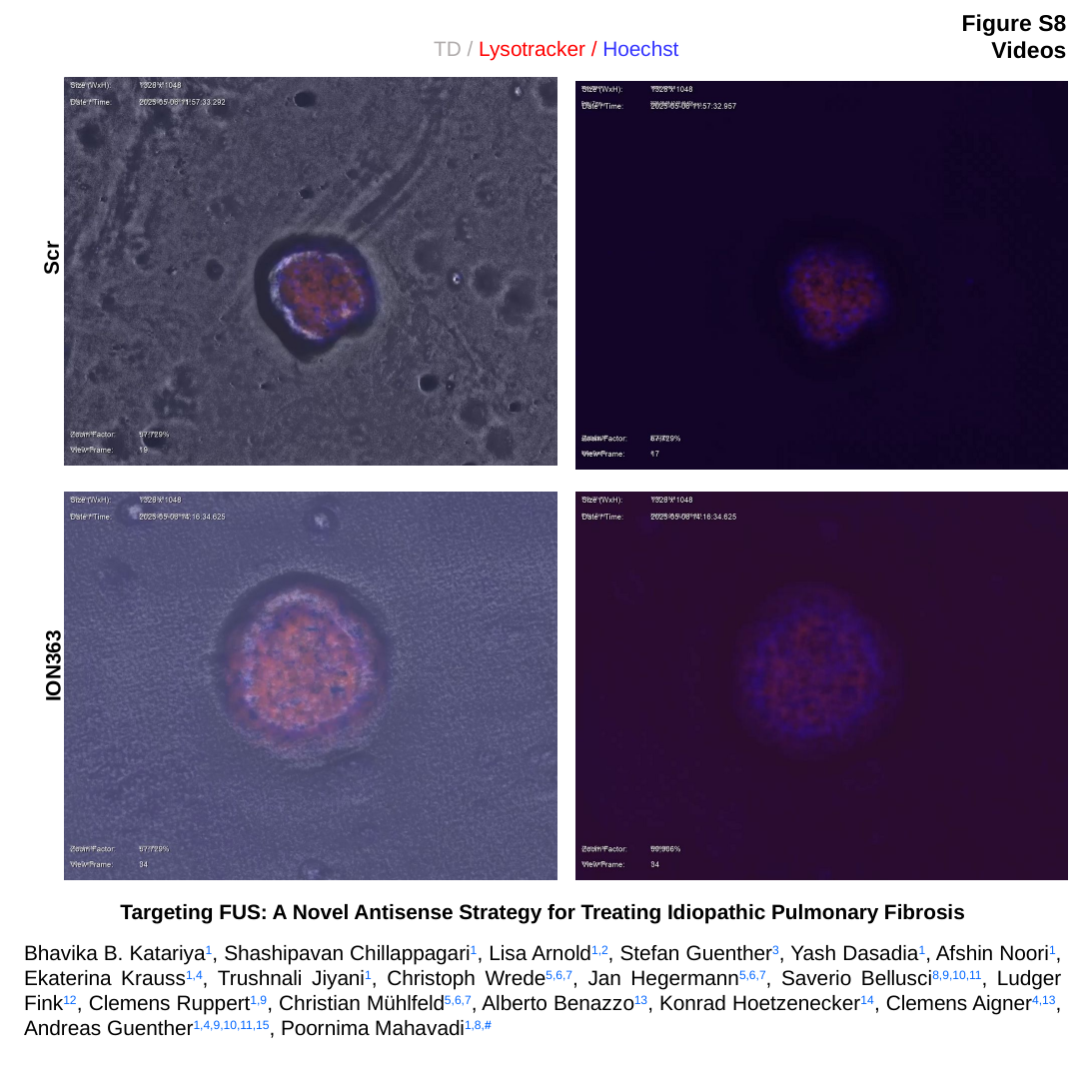

Figure S8
Videos
TD / Lysotracker / Hoechst
Scr
ION363
Targeting FUS: A Novel Antisense Strategy for Treating Idiopathic Pulmonary Fibrosis
Bhavika B. Katariya1, Shashipavan Chillappagari1, Lisa Arnold1,2, Stefan Guenther3, Yash Dasadia1, Afshin Noori1, Ekaterina Krauss1,4, Trushnali Jiyani1, Christoph Wrede5,6,7, Jan Hegermann5,6,7, Saverio Bellusci8,9,10,11, Ludger Fink12, Clemens Ruppert1,9, Christian Mühlfeld5,6,7, Alberto Benazzo13, Konrad Hoetzenecker14, Clemens Aigner4,13, Andreas Guenther1,4,9,10,11,15, Poornima Mahavadi1,8,#
