# Supplementary material for: Targeting fused in sarcoma (FUS): a novel antisense strategy for treating idiopathic pulmonary fibrosis
Source: Signal Transduct Target Ther. 2026 Feb 26;11:70. doi: 10.1038/s41392-026-02585-9 (PMC12936215; doi:10.1038/s41392-026-02585-9)
Supplement: Supplementary file 6 — Supplementary Methods, Tables, references, and figure legends-Clean version [file 41392_2026_2585_MOESM6_ESM.docx]

Supplementary Materials for

Targeting fused in sarcoma (FUS): A novel antisense strategy for treating idiopathic pulmonary fibrosis

Bhavika B. Katariya^1^, Shashipavan Chillappagari^1^, Lisa Arnold^1,2^, Stefan Guenther^3^, Yash Dasadia^1^, Afshin Noori^1^, Ekaterina Krauss^1,4^, Trushnali Jiyani^1^, Christoph Wrede^5,6,7^, Jan Hegermann^5,6,7^, Saverio Bellusci^8,9,10,11^, Ludger Fink^12^, Clemens Ruppert^1,9^, Christian Mühlfeld^5,6,7^, Alberto Benazzo^13^, Konrad Hoetzenecker^14^, Clemens Aigner^4,13^, Andreas Guenther^1,4,9,10,11,15^, Poornima Mahavadi^1,9,*^

Correspondence to: [Poornima.Mahavadi@innere.med.uni-giessen.de](mailto:Poornima.Mahavadi@innere.med.uni-giessen.de)

**This PDF file includes:**

Materials and Methods

Supplementary Text

Figures. S1 to S8

Tables S1 to S3

Captions for Movies S8 / Videos 1-4

**Other Supplementary Materials for this manuscript include the following:**

Movies V1-4

Data S1 to S3

Table S1. Patient information

Table S2. Primers used for q-PCR

Table S3. Antibodies used in this study

Materials and Methods

**RNA extraction & qRT-PCR**

Total RNA was isolated using standard protocols as described before using RNeasy kit from Qiagen according to manufacturer’s instructions and as described before ^1^. Concentration of RNA was measured using Nanodrop. Around 1 µg of RNA was used for cDNA synthesis using the PrimeScript RT Master Mix from Takara Bio Inc. Generated cDNA was diluted 1:10 using sterile RNase free water and equal volumes were used as a template for amplification in a qRT-PCR. Amplification was performed using the primers given in supplementary table 2. Every reaction was performed as duplicates and quantified with the ΔΔC_T_-method. Threshold cycles (C_T_) of target genes were normalized to a housekeeping gene (*ACTB*). The resulting ΔC_T_ were compared to the respective control samples and relative mRNA expression was calculated by R = 2^−ΔΔC^_T_.

**Electron microscopy**

For electron microscopy analysis samples (IPF, n=2; HD, n=2) were prepared as already described^2^. In brief, cells were fixed (4% paraformaldehyde and 0.1% glutaraldehyde in 200 mM Hepes buffer, pH 7.35), embedded in Lowicryl HM20 resin (Polysciences, Inc.) and immunogold labeling was conducted on ultrathin sections with 30 min 3% BSA, primary antibody rabbit anti-FUS IgG (Proteintech) diluted 1:10, secondary antibody goat anti-rabbit IgG 10 nm gold (TED Pella, Inc.) diluted 1:50, as control without primary antibody. No poststain was applied.

**RNA sequencing analysis**

Trimmomatic version 0.39 was employed to trim reads after a quality drop below a mean of Q15 in a window of 5 nucleotides and keeping only filtered reads longer than 15 nucleotides ^3^. Reads were aligned versus Ensembl human genome version hg38 (Ensembl release 109) with STAR 2.7.11a ^4^. Alignments were filtered to remove: duplicates with Picard 3.0.0 (Picard: A set of tools (in Java) for working with next generation sequencing data in the BAM format), multi-mapping, ribosomal, or mitochondrial reads. Gene counts were established with feature Counts 2.0.4 by aggregating reads overlapping exons on the correct strand excluding those overlapping multiple genes ^5^. The raw count matrix was normalized with DESeq2 version 1.36.0 ^6^. Contrasts were created with DESeq2 based on the raw count matrix. Genes were classified as significantly differentially expressed at average count > 5, multiple testing adjusted p-value < 0.05, and -0.585 < log2FC > 0.585. The Ensemble annotation was enriched with UniProt data (Activities at the Universal Protein Resource (UniProt)).

All downstream analyses are based on the normalized gene count matrix. Volcano and MA plots were produced to highlight DEG expression. A global clustering heatmap of samples was created based on the euclidean distance of regularized log transformed gene counts. Dimension reduction analyses (PCA) were performed on regularized log transformed counts using the R packages FactoMineR ^7^ . DEGs were submitted to gene set overrepresentation analyses with KOBAS (Xie et al., KOBAS 2.0: a web server for annotation and identification of enriched pathways and diseases.). The resulting bubble plot shows pathways with Benjamini-Hochberg corrected p-value < 0.05 (represented by dashed line). The larger gray circles are scaled to the number of genes comprising the respective pathway, while the smaller colored circles represent subsets found to be DEGs. Transcription factor binding site (TFBS) enrichment analysis was performed with Pscan ^8^. Reference TFBS position weight matrices CORE_vertebrates_non-redundant_pfms were extracted from JASPAR on 20221124^9^. Overrepresented TFBS were identified based on the promoter nucleotide sequence (450-TSS-50) of protein_coding genes using DEGs as foreground and all genes as background. The resulting heatmap shows TFs that were significantly enriched with uncorrected p-value < 0.05 in at least one foreground list (yellow = overrepresented).

Statistics**:** DESeq2 DE: p-value based on Wald test that was corrected for multiple testing using the Benjamini and Hochberg method Kobas GSO: p-value based on hypergeometric test that was corrected for multiple testing using the Benjamini and Hochberg method.

**Western blot and immunofluorescence**

Denatured cell lysates or lung homogenates were subjected to western blotting following standard protocols as described before ^10,11^ to detect proteins of interest. List of antibodies used can be found in supplementary table 3. Immunofluorescence was performed on primary fibroblasts using standard protocols as described before ^11^ and using antibodies listed in supplementary table 3. Microcopy was performed using a Leica M205 FA fluorescent stereoscope (Leica Microsystems) equipped with a Leica DFC360 FX camera. Image J was used to quantify about 5-6 regions from each group as described before ^11^ and following the instructions in the Image J documentation:

[*https://imagej.net/ij/docs/guide/user-guide.pdf*](https://imagej.net/ij/docs/guide/user-guide.pdf)

**Wound healing assay**

Primary lung fibroblasts were seeded in a 12 well plate at the density of 0.04 X 106 cells/well and kept in CO2 incubator at 37°C for 24h. Following treatments with Scr-ASO (QIAGEN) or ION363 (MedChemExpress) for 48 h, a scratch was made onto the cell monolayer using P200 pipette tip, ensuring straight scratch across all the wells. Cell debris was removed by gently washing cells with cell culture grade 1X DPBS. Later using EVOS phase contrast microscopy, live imaging was performed until 48 hours. Wound area was measured using Image J and percentage wound area was calculated.

**Alveolosphere generation**

Alveolospheres were generated from frozen total cell slurries that were isolated from explanted IPF patient lungs. Tissue dissoaciation and cell slurry preparation as well as 3-dimensional cultures of alveolospheres were performed following protocols that were elegantly described by Katsura et al. and Komishi et.al.^12,13^. Briefly, total cell slurries from explanted IPF patient lungs were prepared by mincing and dissociating approximately 5 g of lung tissue in warm digestion medium and incubating with constant rotations for 1 hour at 37°C. The slurries were then strained through 100 µm cell strainers that were pre-wet with 10%FBS in DMEM/F12 containing anti-anti followed by centrifugation at 1200 g for 10 mins at 4°C. The supernatant was discarded and pellet was dissolved in RBC lysis buffer followed by stopping the lysis by adding 10% FBS in DMEM/F12 containing anti-anti (Miltenyl Biotech). Samples were passed through 40 µm cell strainer. Total cells were cryopreserved in freezing medium (CELLnTEC) at this stage. AT2 cells were isolated as described before^12^ by negative selection using magnet activated cell sorting (MACS) method and as per manufacturer’s instructions using CD31, CD45 micro beads and FcR blocking reagent (all from Miltenyl Biotech). Live cells were counted and about 8000 – 10000 AT2 cells were taken for cytospins to confirm the yield and for quality control purpose by staining them with AT2 markers, HITII-280 and proSP-C (supplementary Fig.S12) and proceeded with the following protocols.

3D culture of human IPF AT2 cells: 24 well plates with inserts (Greiner Bio-One) were prepared. For each insert: 8000 – 10000 AT2 cells + 2000 fibroblasts (primary, healthy) + 50 µL medium + 50 µL matrigel (Corning) was prepared and plated. For ‘alveolospheres alone’ cultures, fibroblasts were omitted. After incubating at 37°C with 5% CO2 for 20 mins, 600 µL of complete medium (Stemm cell technologies) was added to the lower chamber and 400 µL of medium was added inside the insert. 3-4 days later, medium was replaced with fresh medium containing either Scr-ASO or ION363 and every 3-day treatment regime was followed. Lysotracker^TM^ Red DND-99 (Thermo Fisher) was added to the inserts as per manufacturer’s instructions and incubated for 45 – 60 minutes minutes at 37°C. Later, this was replaced with fresh medium and live imaging of alveolospheres were performed. EVOS M7000 (Thermo Fisher) with incubator (37°C, 5% CO2) was used for imaging the alveolospheres and z-stacks were procured. Celleste 6.0 software was used for image analysis, quantifications and videos.

Whole mount immunofluorescence was performed following protocols available here: <https://www.stemcell.com/performing-icc-staining-epithelial-organoids.html#part-3>

Briefly, matrigel was dissolved using cell recovery solution (Corning) as per manufacturer’s instructions and following brief centrifugation, alveolospheres were fixed in 4% paraformaldehyde. Following washing, antigen retrieval was performed using citrate buffer (pH 6.0) at 98°C, permeablized with 0.3 M glycine followed by blocking with Cytoblock (Invitrogen). Primary antibodies were then added and incubated overnight at 4°C. Next day, alveolospheres were washed and incubated with Alexa Fluor labelled secondary antibodies for 2 hours followed by nuclei staining with Hoechst (Cell Signaling). Samples were washed with PBS and were imaged using Olympus FV 3000 microscope and Z-stacks were aquired. Imaris 8.0 software was used to visualize and process the acquired images and vidoes.


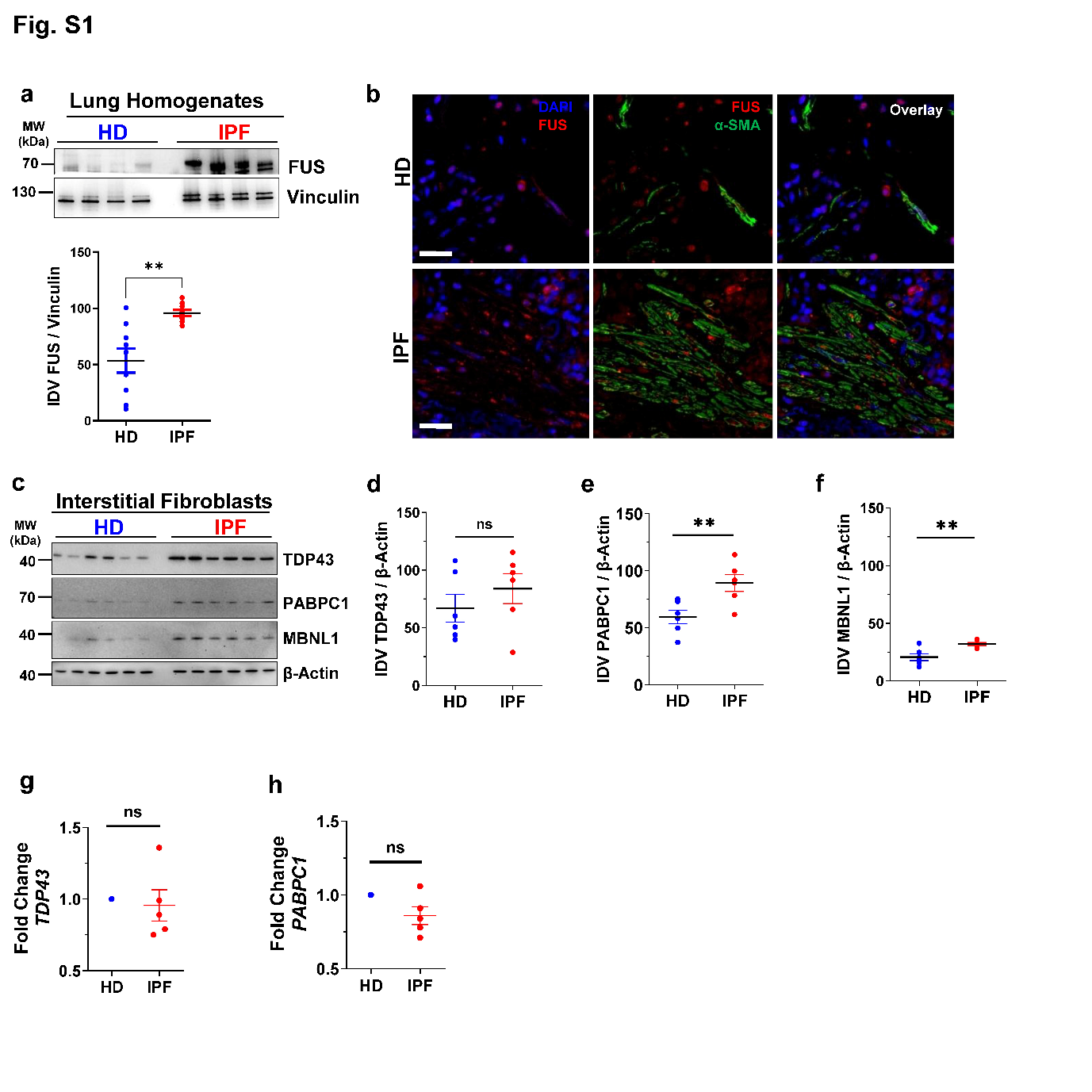


Figure. S1.

(**a**) Immunoblot analysis of FUS or β-Actin from total lung homogenates derived from the lungs of healthy donors (HD) or IPF patients. Quantification after normalizing the integrated Density Values (IDV) of FUS to β-Actin. Blots and analysis from n=8 HD and n=8 IPF patient fibroblasts. (**b**) Immunofluorescence analysis for FUS (red) and the fibroblast marker, α-smooth muscle actin (α-SMA, green) on lung tissue sections of HD and IPF patient lungs. DAPI was used for nuclear staining. Image J was used for quantification and analysis was performed from n=3 HD & n=3 IPF patients. Scale bar = 25 µm. (**c**) Immunoblot analysis of TDP43, PABPC1 and MBNL1 or β-Actin from total lysates of fibroblasts derived from the lungs of HD or IPF patients. (**d-f**) Quantification after normalizing the IDVs of the indicated proteins to β-Actin. Blots and analysis from n=6 HD and n=6 IPF patient fibroblasts. (**g and h**) Analysis of TDP43, PABPC1 mRNA using qRT-PCR in fibroblasts derived from HD or IPF lungs. Values were normalized to the housekeeping gene β-Actin (ACTB). FUS mRNA expression in HD fibroblasts was set as one. P value: **P<0.01, ns = not significant.


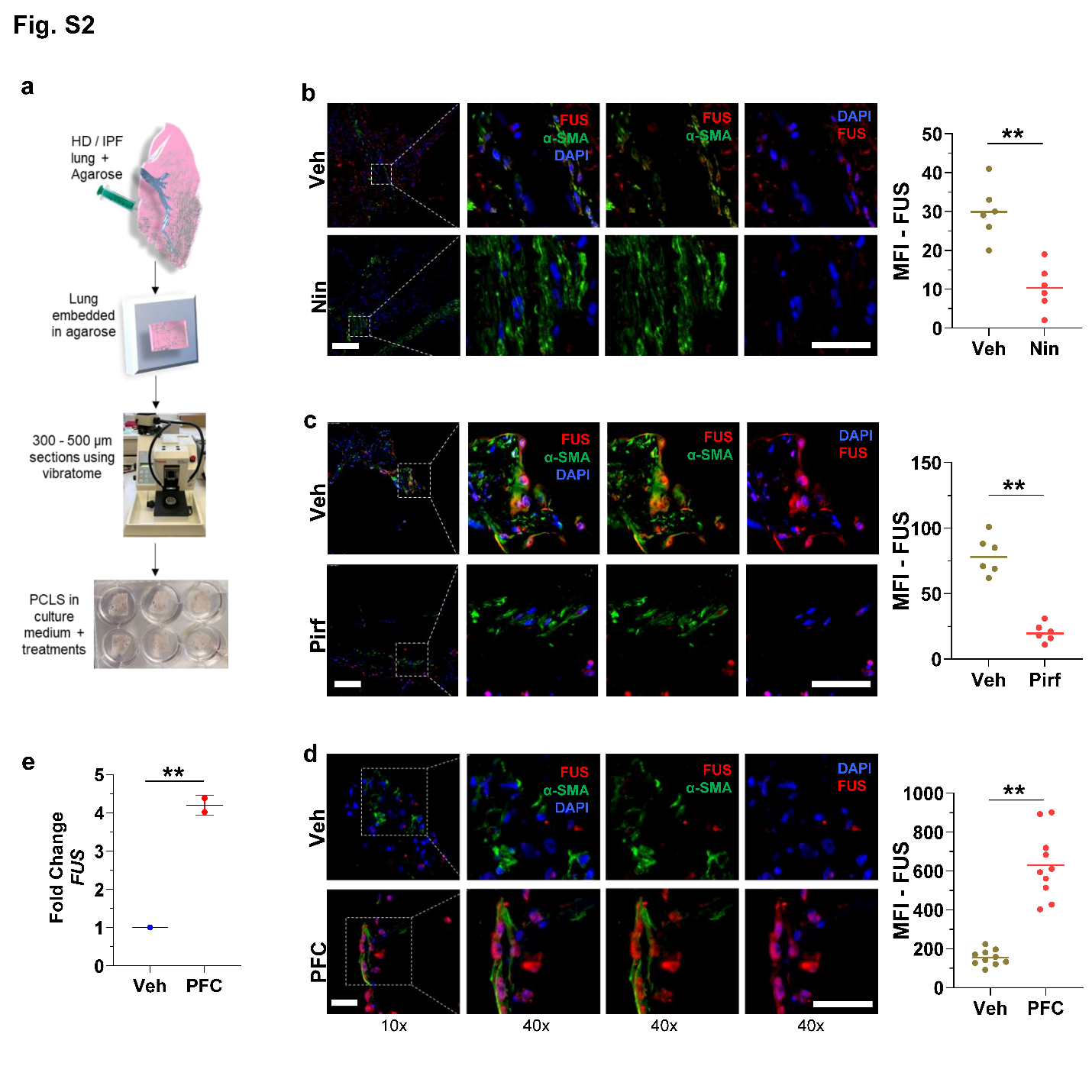


Figure. S2.

(**a**) Scheme depicting the workflow involved in the generation of PCLS. (**b-d**) IPF PCLS were treated either with Veh, Nintedanib (Nin; n=3 patients / group) or Pirfenidone (Pirf; n=2 patients / group) for 24h (b&c) or HD PCLS were treated with vehicle (Veh) or pro-fibrotic cocktail (PFC). for 48h (n=2 HD / group, followed by fixation in formalin and embedding in paraffin. 3 – 5 µm sections were performed and were stained for FUS (red) or α-SMA (green). DAPI was used for staining the nuclei. On the right side, graphs represent mean fluorescence intensity (MFI) of FUS staining that was quantified using ImageJ. Scale bars in b, c, d for 10x images = 20 µm; for 40 x images = 50 µm. (**e**) Analysis of FUS mRNA using qRT-PCR from HD fibroblasts treated with Veh or PFC. P value: **P<0.01.


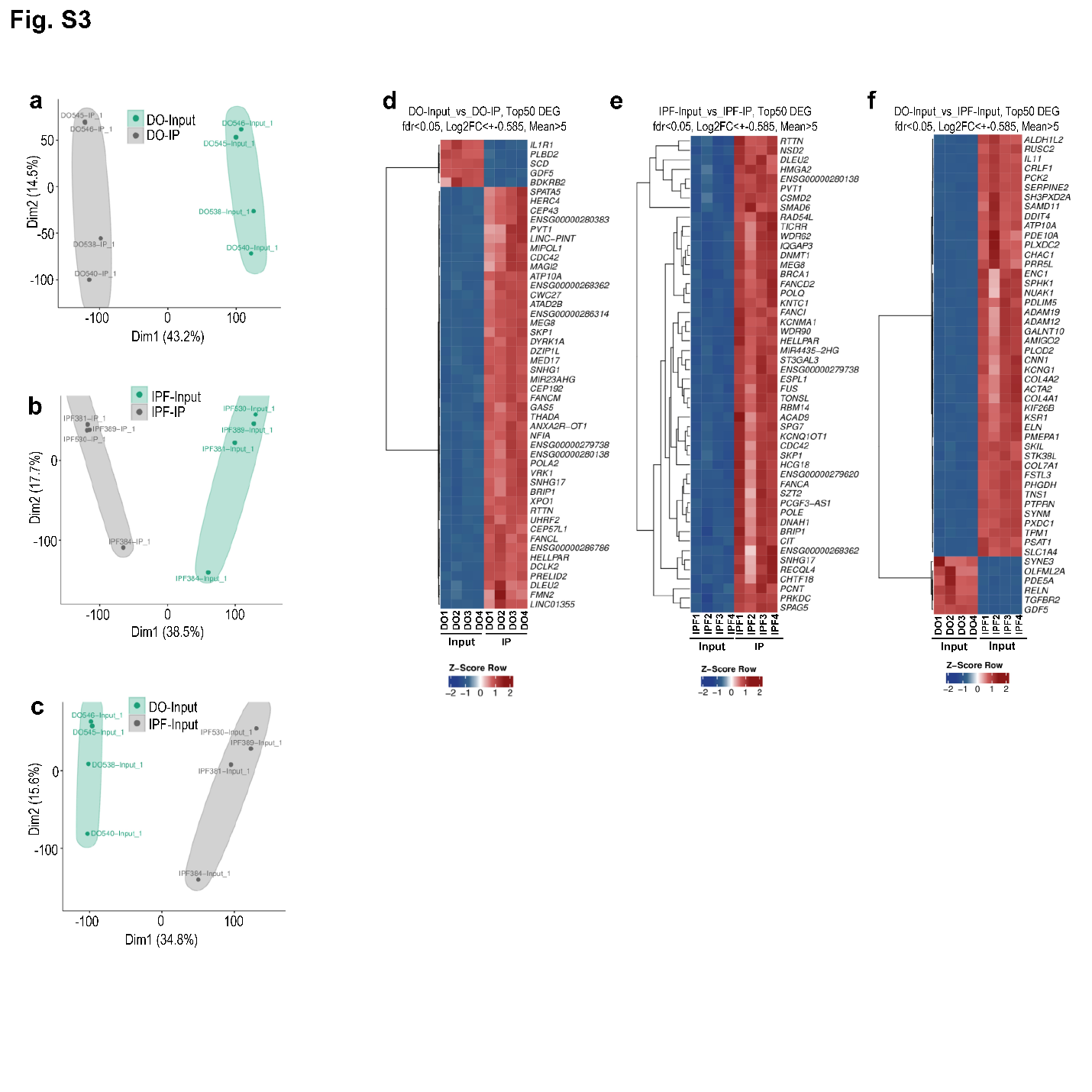


Figure. S3.

(**a,b,c**). PCA of the data sets of the indicated groups is shown. (**d,e,f**) Z score heatmap plots of the top 50 DEGs that were enriched in Do-IP vs Do-Input (d), IPF-IP vs. IPF-Input (e) and IPF-Input vs Do-Input (f). Experiments and analyses were performed on HD and IPF fibroblasts (n=4 each).


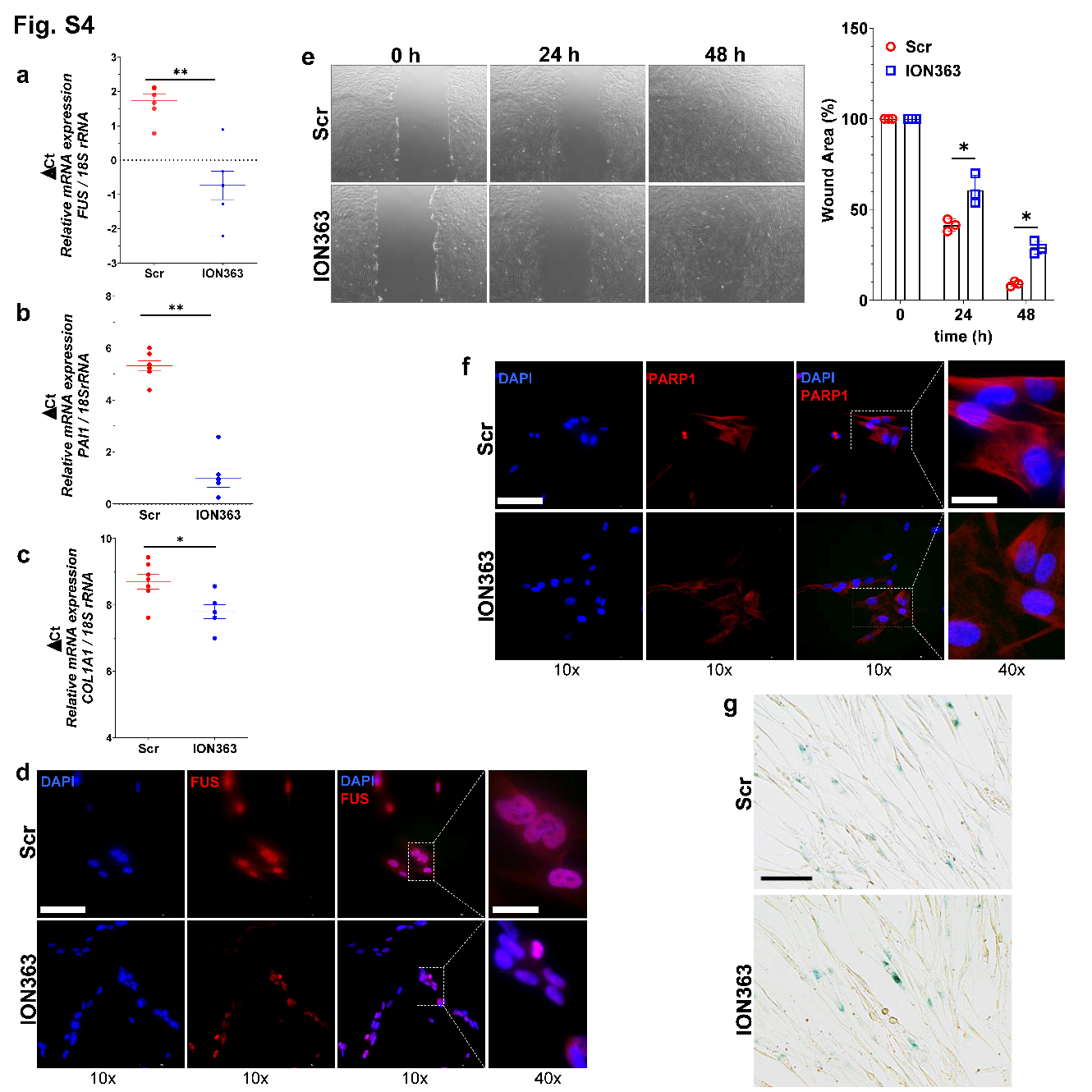


Figure. S4.

Analysis of (**a**) FUS, (**b**) PAI-1 (**c**) COL1A1 mRNA using qRT-PCR from IPF fibroblasts treated with Scr or ION363. P value: **P<0.01, *P<0.05. (**d**) IPF fibroblasts were treated either with scramble ASO (Scr) or ION363 followed by immunofluorescence for FUS (red). (**e**) Wound healing assay to determine cell migration was performed in IPF fibroblasts treated with Scr or ION363. Representative phase-contrast microscope images showing the area covered by the cells at 0, 24 and 48 hours and % wound area was measured. Analysis from n=3 IPF fibroblasts. *P<0.05. (**f**) Immunofluorescence of IPF fibroblasts for cleaved PARP1 (red) following Scr or ION363 treatments. Scale bar for 10x images = 20 µm, for 40x images = 50 µm. (**g**) Representative pictures of senescence associated – β-galactosidase staining in IPF fibroblasts treated with Scr or ION363. Scale bar = 50 µm.


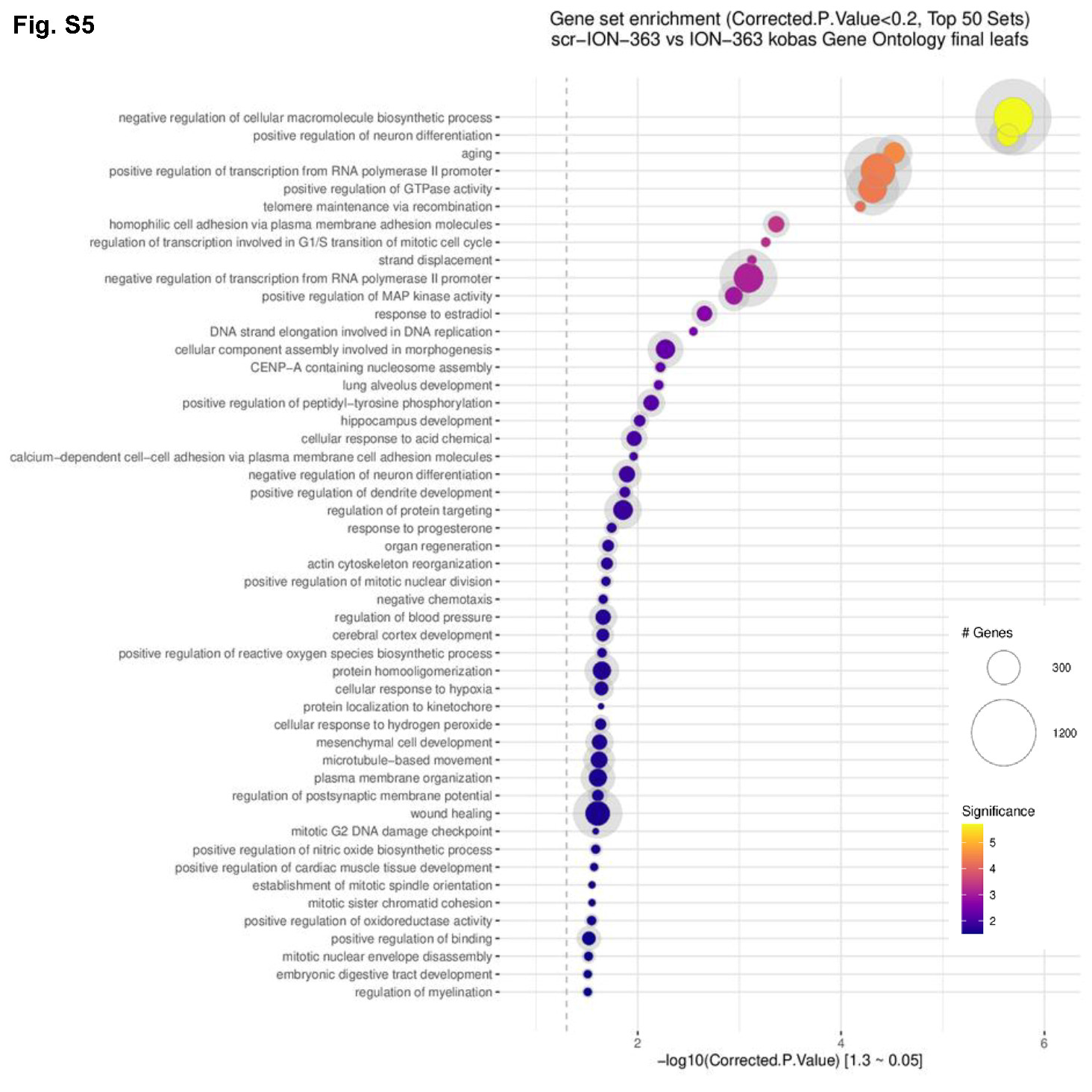


Figure. S5.

IPF fibroblasts were treated with ION363 (15 µg) for 48 hours followed by RNA extraction, RNA-seq and analysis. Several databases were used to analyze our data. Shown here are gene set enrichment analysis of top 50 sets using kobas for GO (final leafs) database. Analysis as indicated in main figure 4 from fibroblasts derived from n=4 IPF patients.


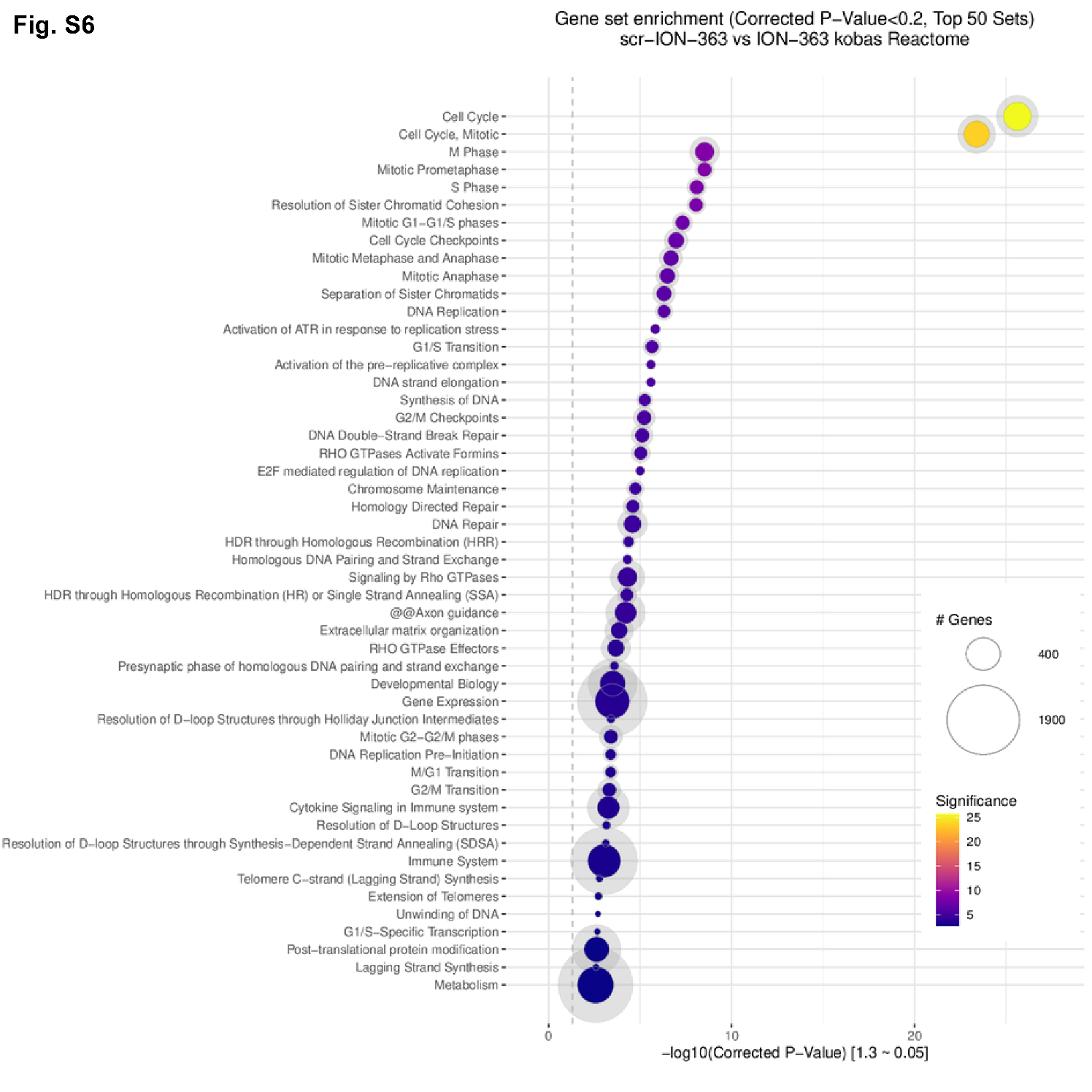


Figure. S6.

IPF fibroblasts were treated with ION363 (15 µg) for 48 hours followed by RNA extraction, RNA-seq and analysis. Several databases were used to analyze our data. Shown here are gene set enrichment analysis of top 50 sets using kobas for Reactome database. Analysis as indicated in main figure 4 from fibroblasts derived from n=4 IPF patients.


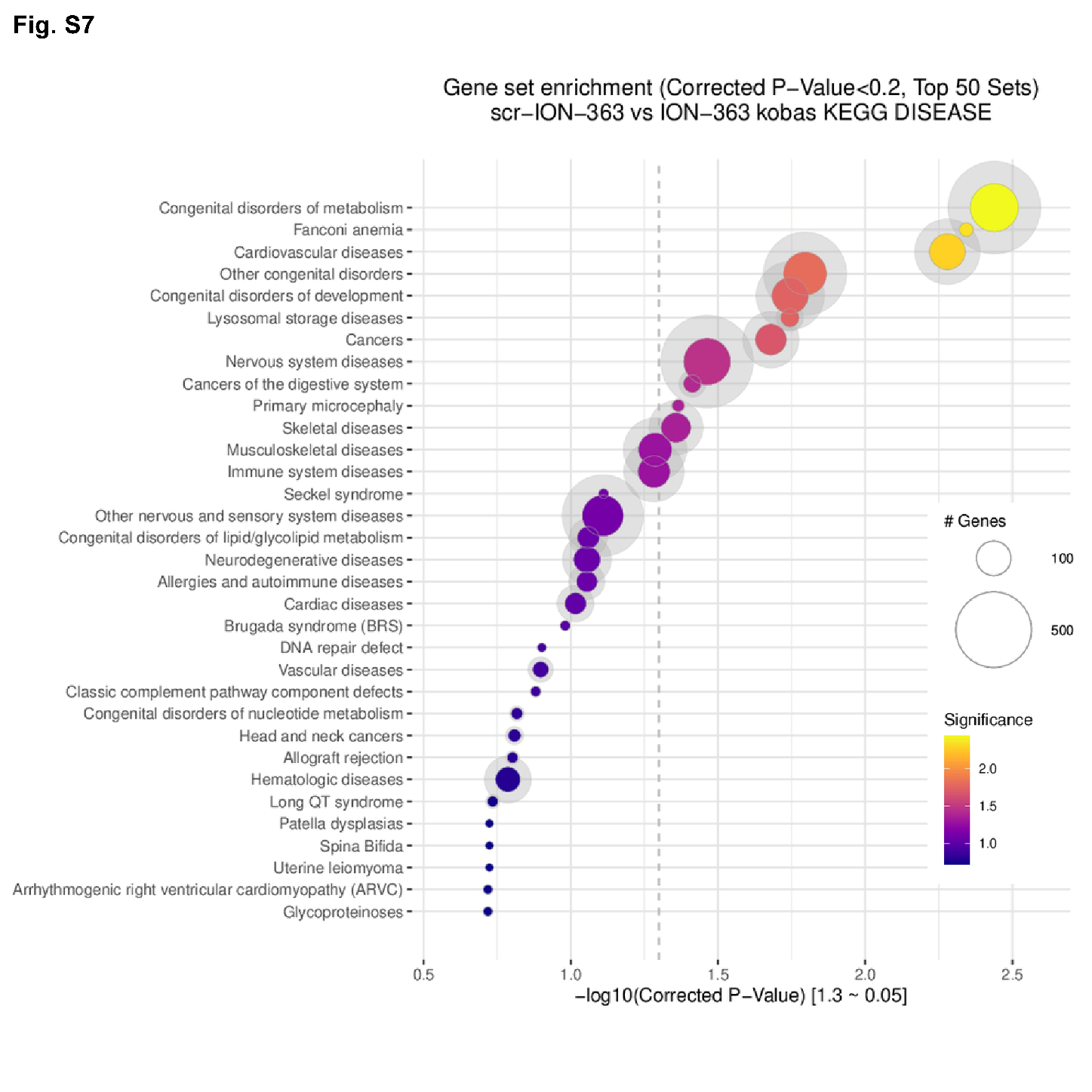


Figure. S7.

IPF fibroblasts were treated with ION363 (15 µg) for 48 hours followed by RNA extraction, RNA-seq and analysis. Several databases were used to analyze our data. Shown here are gene set enrichment analysis of top 50 sets using kobas for KEGG disease database. Analysis as indicated in main figure 4 from fibroblasts derived from n=4 IPF patients.


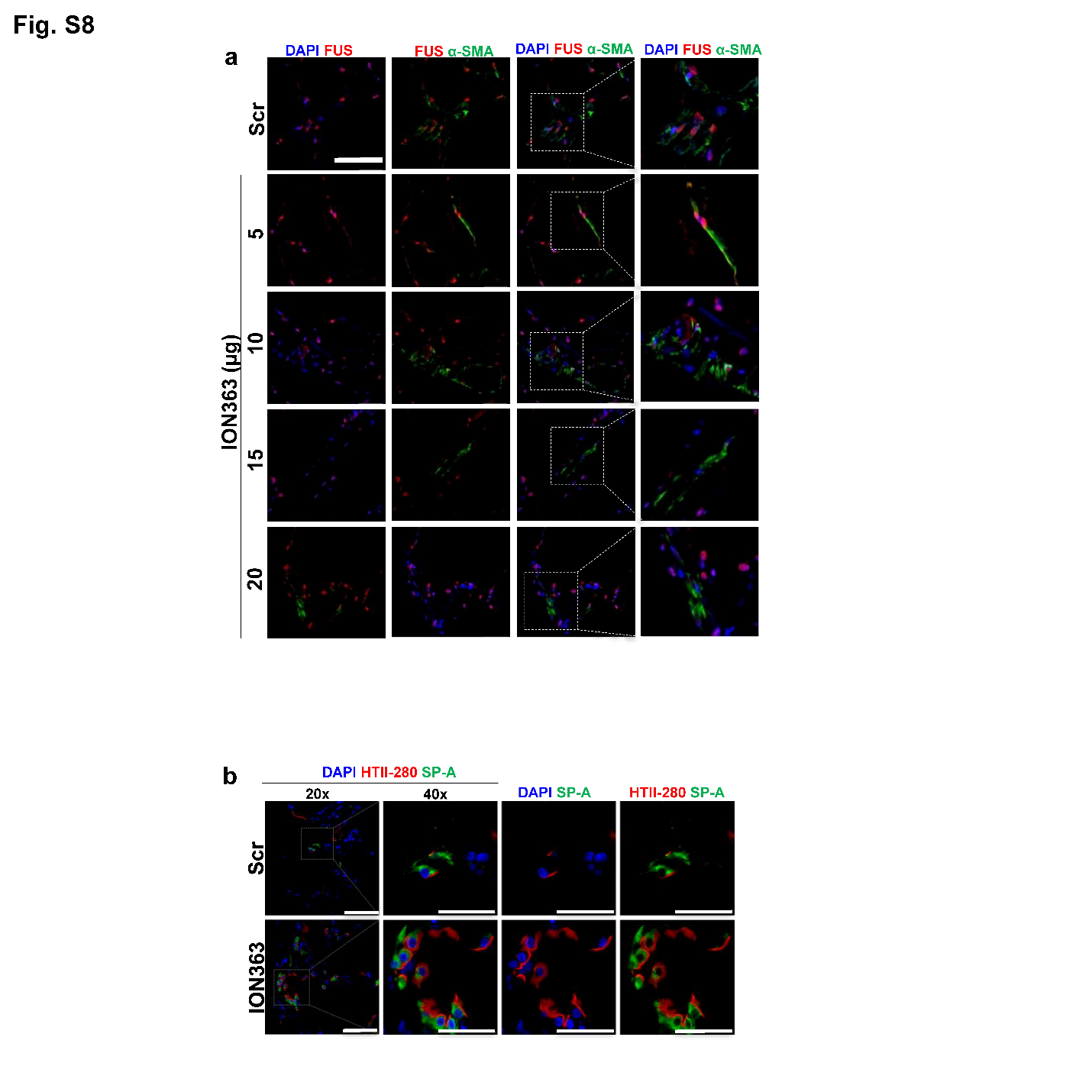


Figure. S8.

(**a**) IPF PCLS were treated either with scramble ASO (Scr) or different concentrations of ION363 i.e. 5 µg, 10 µg, 15 µg, 20 µg followed by their fixation in formalin and embedding in paraffin. 3 µm sections were performed and were stained for FUS (red) or α-SMA (green). DAPI was used for staining the nuclei. Graph represents fluorescence intensity of FUS staining that was quantified using ImageJ. Analysis was performed from n=3 IPF patients. Scale bar = 10 µm. (**b**) Immunofluorescence analysis of HTII-280 (red) and surfactant protein A (SP-A, green) in IPF PCLS treated with Scr or ION363. Scale bar in 20x images = 20 µm, in 40x images = 100 µm.


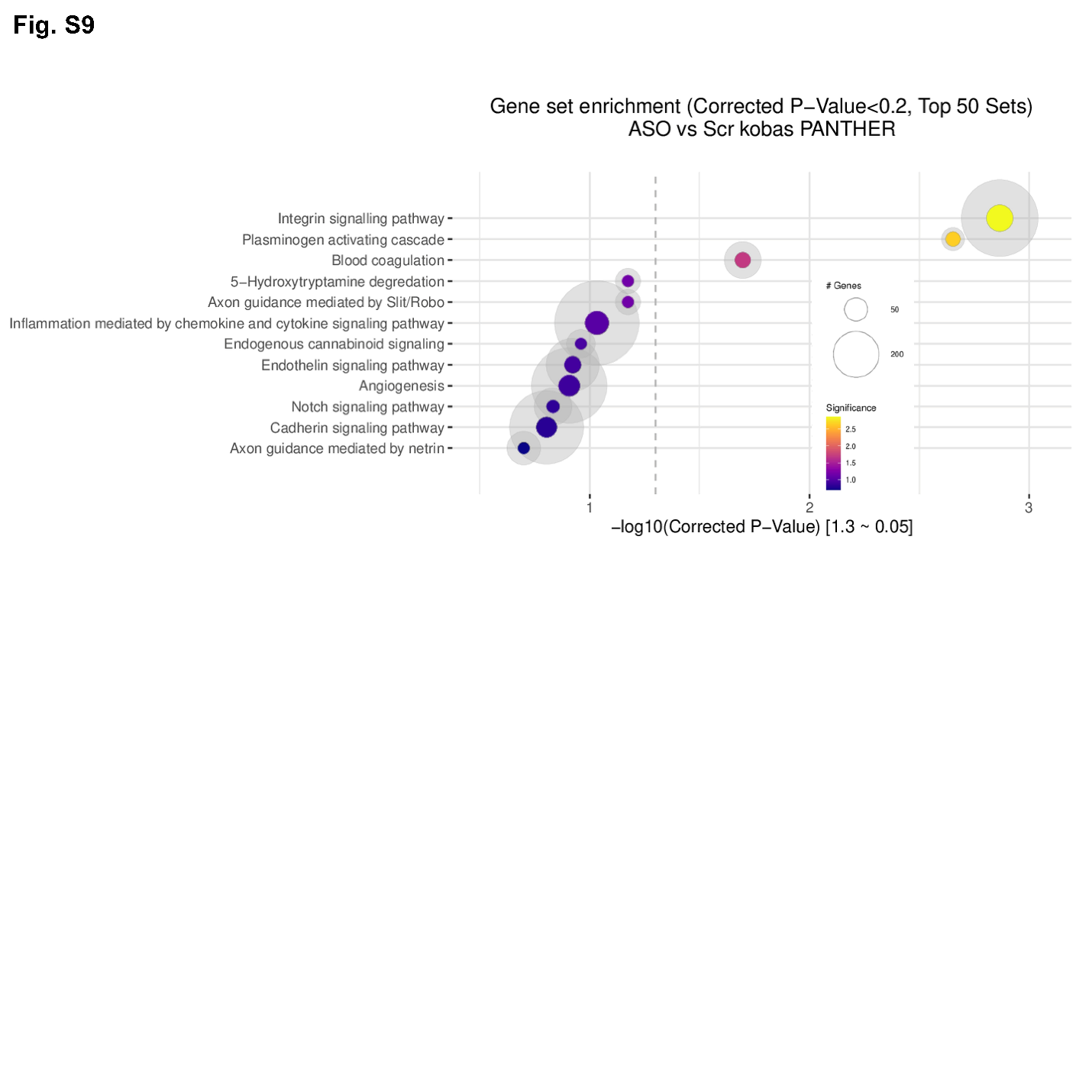
Figure. S9.

IPF PCLS were treated either with scramble ASO (Scr) and processed as indicated for main Fig. 6. Gene set enrichment analysis of the top 50 sets using kobas for PANTHER database showing genes of the integrin signaling and coagulation cascade to be differentially regulated.


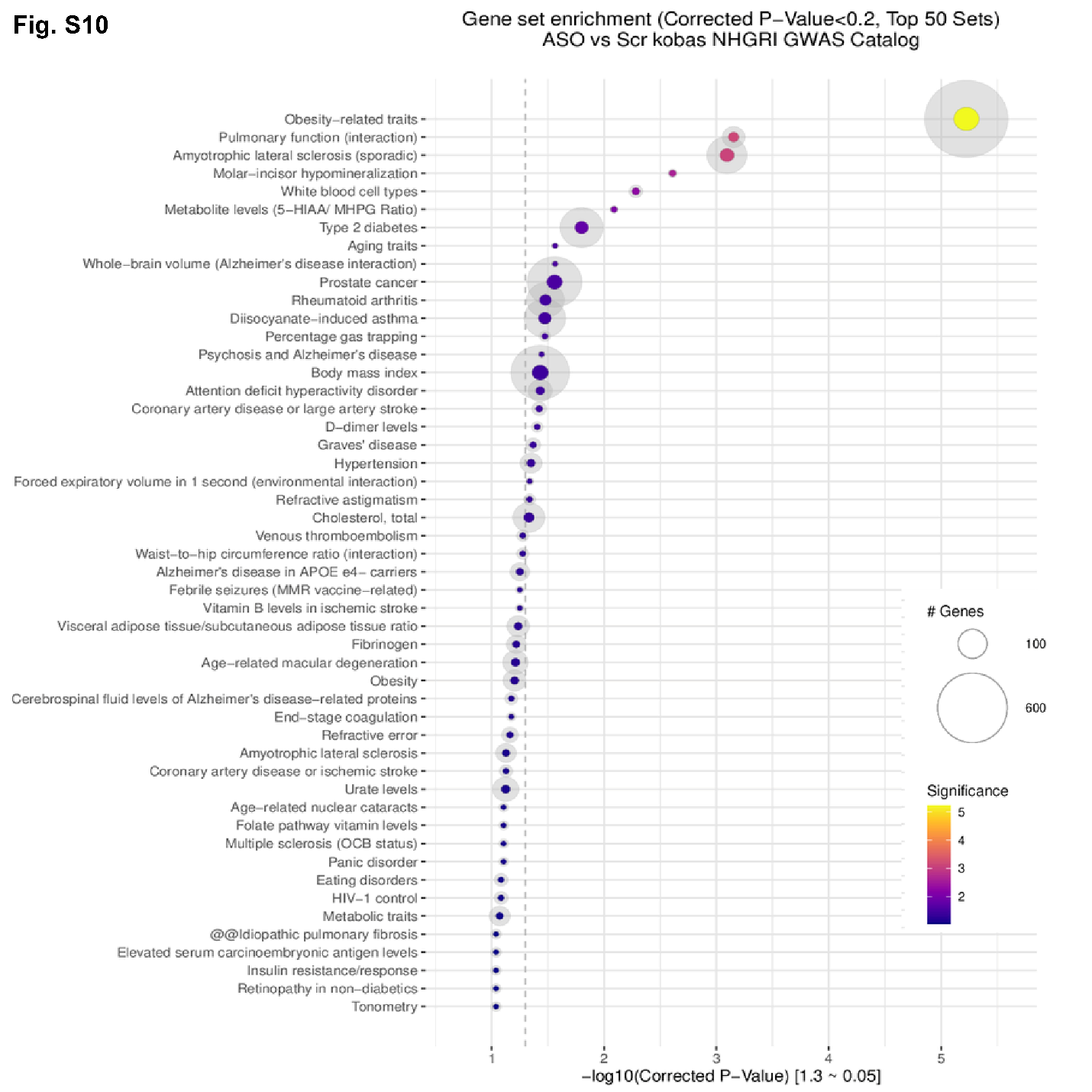


Figure. S10.

IPF PCLS were treated either with scramble ASO (Scr) and processed as indicated for main Fig. 6. Gene set enrichment analysis of the top 50 sets using kobas for for NHGRI GWAS database is shown.


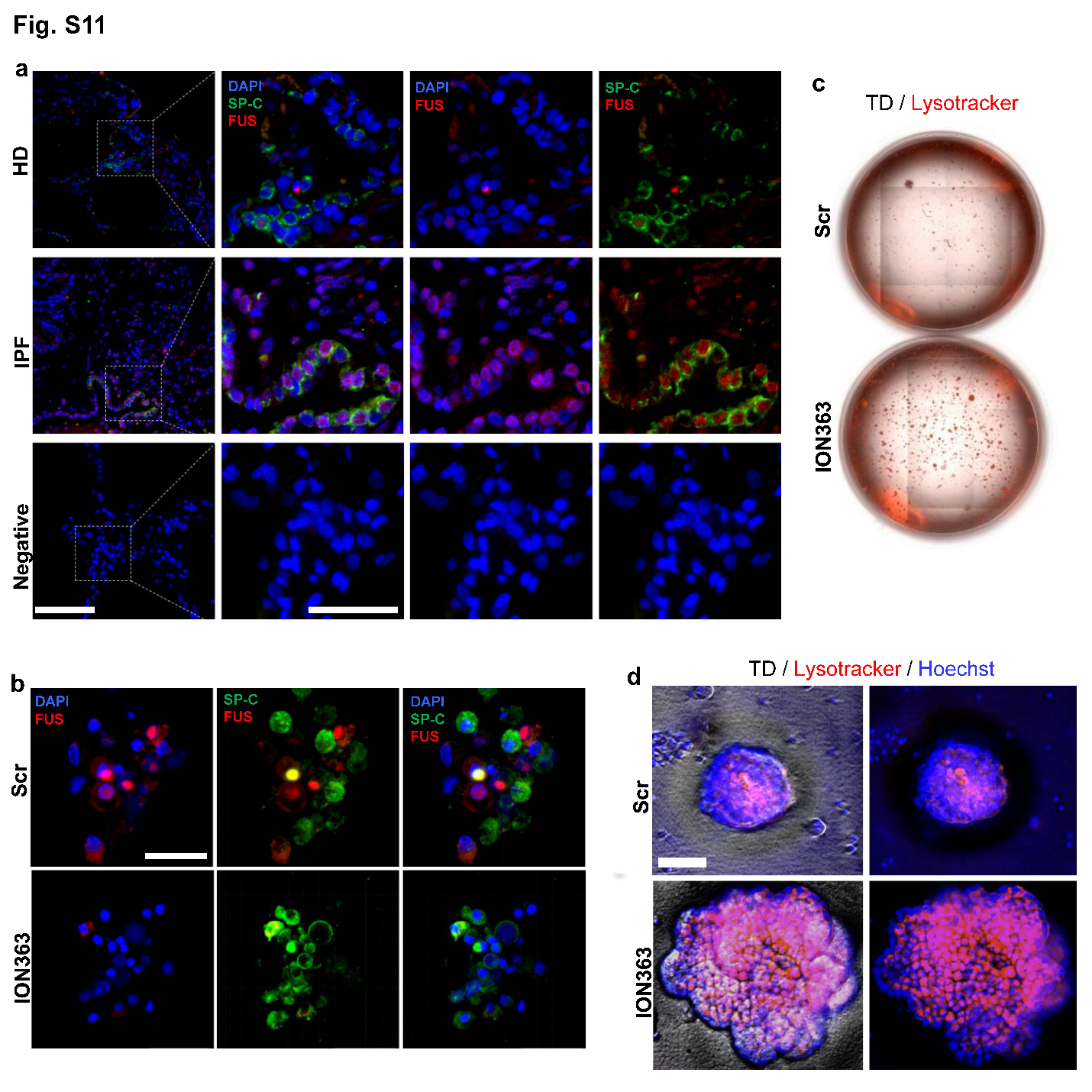


Figure. S11.

Representative immunofluorescence images of (a) paraffin embedded IPF and HD lung sections or (b) primary AT2 cells isolated from IPF patient lungs stained for pro SP-C (green), FUS (red) and DAPI (blue). Scale bar = 20 µm for images in the far left panel. Scale bar = 100 µm for all other images, (c) Alveolospheres treated with Scr-ASO (Scr) or ION363 were until day 14 were stained with lysotracker red to stain acidic compartments, followed by live imaging at 4x in EVOSM7000. Stitched image with TD (transmitted light / bright field) was saved using Celleste 6.0. (d) Representative images of one 3D live alveolosphere stained with lysotracker / hoechst and imaged using EVOS M7000 at 20x from n = 3 IPF patients. Scale bar = 20 µm.


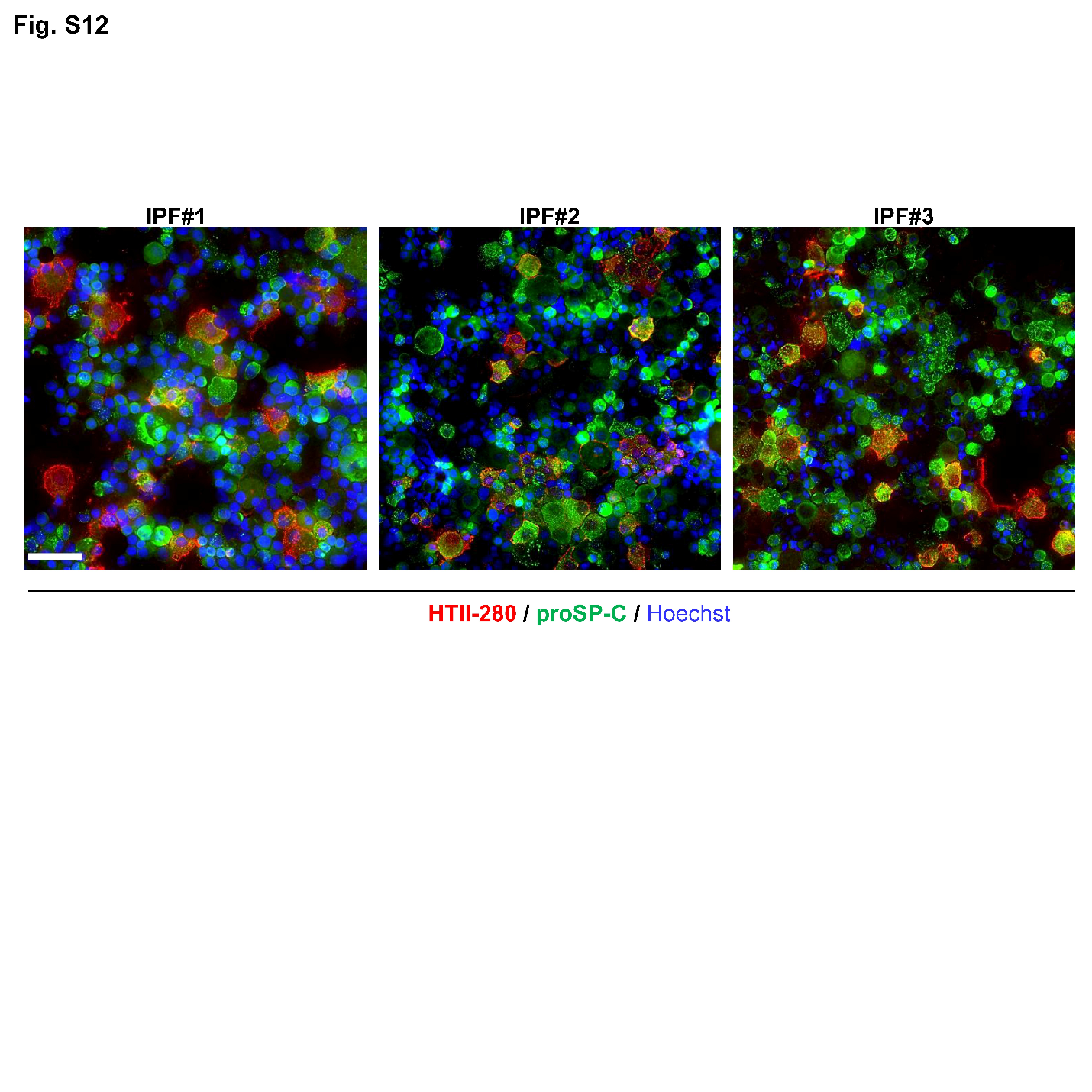


Figure. S12.

Representative immunofluorescence images of primary AT2 cells isolated from cell slurries of IPF lungs. Shown here is the staining of AT2 cell markers, HTII-280 in red and proSP-C in green on cytospins performed from freshly isolated AT2 cells, a quality control experiment for the purity of AT2 cells. Nuclei are stained with DAPI. Scale bar = 50 µm. Representative images of AT2 cells isolated from n = 3 IPF patients are shown here.


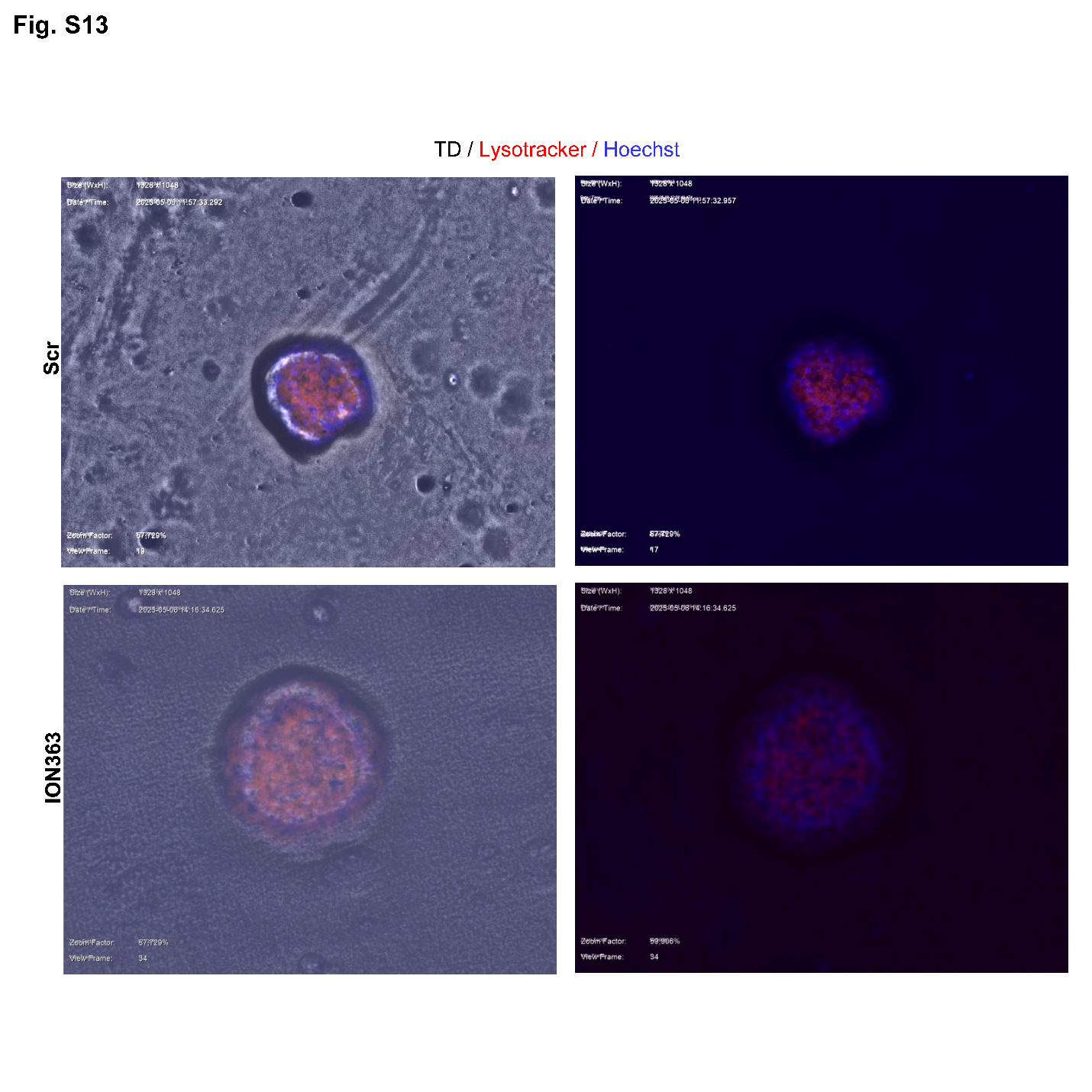
Figure. S13

Representative images / thumbnails of vidoes of 3D alveolospheres shown in main Figure 8d, full videos given in Videos V1 – V4. The videos depict an increase in size of alveolospheres and an increase in lysotracker uptake by alveolospheres treated with ION363. 3D imaging was performed from alveolospheres generated from AT2 cells isolated from n=3 IPF patients.

Video. V1 Alveolospheres treated with Scr ASO-All Channels.

Video. V2 Alveolospheres treated with Scr ASO-Lysotracker-Red, Hoechst-Blue.

Video. V3 Alveolospheres treated with FUS-ASO, ION363-All Channels.

Video. V4 Alveolospheres treated with FUS-ASO, ION363-Lysotracker-Red, Hoechst-Blue.

| **Patient** | **Age** | **Gender** | **Total lung homogenates** | **Primary fibroblasts** | **PCLS** | **Lung Tissue Sections** | **Alveolo-spheres** |
| --- | --- | --- | --- | --- | --- | --- | --- |
| Do#1 | 48 | M | ✓ | - | - | - | - |
| Do#2 | 52 | F | ✓ | - | - | - | - |
| Do#3 | 51 | F | ✓ | - | - | - | - |
| Do#4 | 60 | F | ✓ | - | - | - | - |
| Do#5 | - | - | ✓ | - | - | - | - |
| Do#6 | - | - | ✓ | - | - | - | - |
| IPF#1 | 73 | F | ✓ | - | - | - | - |
| IPF#2 | 62 | M | ✓ | - | - | - | - |
| IPF#3 | 62 | M | ✓ | - | - | - | - |
| IPF#4 | 68 | M | ✓ | - | - | - | - |
| IPF#5 | - | - | ✓ | - | - | - | - |
| IPF#6 | - | - | ✓ | - | - | - | - |
| Do#7 | 60 | M | - | ✓ | - | - | - |
| Do#8 | 72 | F | - | ✓ | - | - | - |
| Do#9 | 37 | F | - | ✓ | - | - | - |
| Do#10 | 58 | F | - | ✓ | - | - | - |
| Do#11 | 59 | F | - | ✓ | - | - | - |
| Do#12 | 31 | F | - | ✓ | - | - | - |
|  |  |  |  |  |  |  |  |
| Do#13 | 54 | M | - | ✓ | - | - | - |
| IPF#7 | 48 | F | - | ✓ | - | - | - |
| IPF#8 | 38 | M | - | ✓ | - | - | - |
| IPF#9 | 61 | M | - | ✓ | - | - | - |
| IPF#10 | 56 | M | - | ✓ | - | - | - |
| IPF#11 | 53 | F | - | ✓ | ✓ | - | - |
| IPF#12 | 55 | M | - | ✓ | - | - | - |
| IPF#13 | 65 | M | - | ✓ | - | - | - |
| Do#14 | 51 | - | - | - | ✓ | - | - |
| Do#15 | 49 | - | - | - | ✓ | - | - |
| IPF#14 | 52 | F | - | - | ✓ | - | - |
| IPF#15 | 70 | M | - | - | ✓ | - | - |
| IPF#16 | 61 | M | - | - | ✓ | - | - |
| IPF#17 | 59 | M | - | - | ✓ | - | - |
| Do#16 | 30 | M | - | - | - | ✓ | - |
| Do#17 | - | - | - | - | - | ✓ | - |
| Do#18 | 46 | F | - | - | - | ✓ | - |
| Do#19 | - | - | - | - | - | ✓ | - |
| Do#20 | 21 | F | - | - | - | ✓ | - |
| Do#21 | 26 | F | - | - | - | ✓ | - |
| Do#18 | 16 | M | - | - | - | ✓ | - |
| IPF#19 | 63 | M | - | - | - | ✓ | - |
| IPF#20 | 57 | F | - | - | ✓ | ✓ | - |
| IPF#21 | 63 | M | - | - | - | ✓ | - |
| IPF#22 | 54 | F | - | - | ✓ | ✓ | - |
| IPF#23 | 61 | M | - | - | - | ✓ | - |
| IPF#24 | 53 | F | - | - | ✓ | ✓ | - |
| IPF#25 | 32 | F | - | - | - | ✓ | - |
| IPF#26 | 60 | F | - | - | - | - | ✓ |
| IPF#27 | 50 | M | - | - | - | - | ✓ |
| IPF#28 | 71 | M | - | - | - | - | ✓ |
| IPF#29 | 61 | M | - | - | - | - | ✓ |
| IPF#30 | 58 | F | - | - | - | - | ✓ |
| IPF#31 | 63 | M | - | - | - | - | ✓ |

Table S1.

Patient information

| **Gene name** | **Sequence** |
| --- | --- |
| *FUS* | 5'- CAG ACA GGG AAA CTG GCA AGC T -3'  5'- GGC GAG TAG CAA ATG AGA CCT TG -3' |
| *TDP43* | 5'- GAT GGA CGA TGG TGT GAC TGC A -3  5'- AAG AAC TCC CGC AGC TCA TCC T -3' |
| *PABPC1* | 5'- AGT CAC TCC GTT CTA AGG TTG A -3  5'- GCA CAA GTT TCT TTT CAT GGT CC -3 |
| *GAPDH* | 5'- GTC TCC TCT GAC TTC AAC AGC G -3'  5'- ACC ACC CTG TTG CTG TAG CCA A -3' |
| *β-ACTIN* | 5'- ACC CTG AAG TAC CCC ATC G -3'  5'- CAG CCT GGA TAG CAA CGT AC -3' |
| *18sRNA* | 5'- ACC CGT TGA ACC CCA TTC GTG A -3'  5'- GCC TCA CTA AAC CAT CCA ATC GG -3' |
| *PAI-1* | 5' CTC ATC AGC CAC TGG AAA GGC A 3'  5' GAC TCG TGA AGT CAG CCT GAA AC 3' |
| *COL1A1* | 5' GAT TCC CTG GAC CTA AAG GTG C 3'  5' AGC CTC TCC ATC TTT GCC AGC A 3' |

Table S2.

Primers used for q-PCR

| **Name** | **Company** | **WB** | **EM** | **IP** | **IF** |
| --- | --- | --- | --- | --- | --- |
| FUS | Proteintech | ✓ | ✓ | ✓ | ✓ |
| TDP43 | Proteintech | ✓ | - | - | ✓ |
| PABPC1 | Proteintech | ✓ | - | - | ✓ |
| MBNL1 | Invitrogen | ✓ | - | - | ✓ |
| TIA1 | Abcam | ✓ | - | - | ✓ |
| G3BP1 | Novus | ✓ | - | - | ✓ |
| GAPDH | Cell signalling | ✓ | - | - | - |
| ß-ACTIN | Abcam | ✓ | - | - | - |
| ACTA2 | Merck | - | - | - | ✓ |
| ACTA2 | Abcam | - | - | - | ✓ |
| Vinculin | Merck | ✓ | - | - | - |
| Turbo-GFP | Origene | ✓ | - | - | - |
| PCNA | Santa cruz | ✓ | - | - | - |
| HTII-280 | Terrace Biotech | - | - | - | ✓ |
| AQP5 | Santa Cruz | - | - | - | ✓ |
| COL1A1 | Rockland Immunochemicals | - | - | - | ✓ |
| SP-A | Proteintech | - | - | - | ✓ |
| Cleaved PARP1 | abcam | - | - | - | ✓ |
| Pro SP-C | Millipore | - | - | - | ✓ |

**Table S3.** Antibodies used in this study.

Video V1.

Representative vidoes of 3D alveolospheres shown in figure 7d. The videos depict an increase in size of alveolospheres and an increase in lysotracker uptake by alveolospheres treated with ION363. 3D imaging was performed from alveolospheres generated from AT2 cells isolated from n=3 IPF patients.

Supplementary References.

1. Chillappagari et al. SIAH2-mediated and organ-specific restriction of HO-1 expression by a dual mechanism. Scientific reports. 10, 2268 (2020).

2. Eggenschwiler et al. A combined in silico and in vitro study on mouse Serpina1a antitrypsin-deficiency mutants. Sci Rep. 9, 7486 (2019).

3. Bolger et al. Trimmomatic: a flexible trimmer for Illumina sequence data. Bioinformatics. 30, 2114-2120 (2014).

4. Dobin et al. STAR: ultrafast universal RNA-seq aligner. Bioinformatics. 29, 15-21 (2013).

5. Liao et al. featureCounts: an efficient general purpose program for assigning sequence reads to genomic features. Bioinformatics. 30, 923-930 (2014).

6. Love et al. Moderated estimation of fold change and dispersion for RNA-seq data with DESeq2. Genome Biol. 15, 550 (2014).

7. Le et al. FactoMineR: An R package for multivariate analysis. J Stat Softw. 25, 1-18 (2008).

8. Zambelli et al. Pscan: finding over-represented transcription factor binding site motifs in sequences from co-regulated or co-expressed genes. Nucleic Acids Res. 37, W247-252 (2009).

9. Castro-Mondragon et al. JASPAR 2022: the 9th release of the open-access database of transcription factor binding profiles. Nucleic Acids Res. 50, D165-D173 (2022).

10. Korfei et al. Aberrant expression and activity of histone deacetylases in sporadic idiopathic pulmonary fibrosis. Thorax. 70, 1022-1032 (2015).

11. Kesireddy et al. Susceptibility of microtubule-associated protein 1 light chain 3beta (MAP1LC3B/LC3B) knockout mice to lung injury and fibrosis. FASEB journal : official publication of the Federation of American Societies for Experimental Biology. 33, 12392-12408 (2019).

12. Katsura et al. Human Lung Stem Cell-Based Alveolospheres Provide Insights into SARS-CoV-2-Mediated Interferon Responses and Pneumocyte Dysfunction. Cell Stem Cell. 27, 890-904 e898 (2020).

13. Konishi et al. Defined conditions for long-term expansion of murine and human alveolar epithelial stem cells in three-dimensional cultures. STAR Protoc. 3, 101447 (2022).





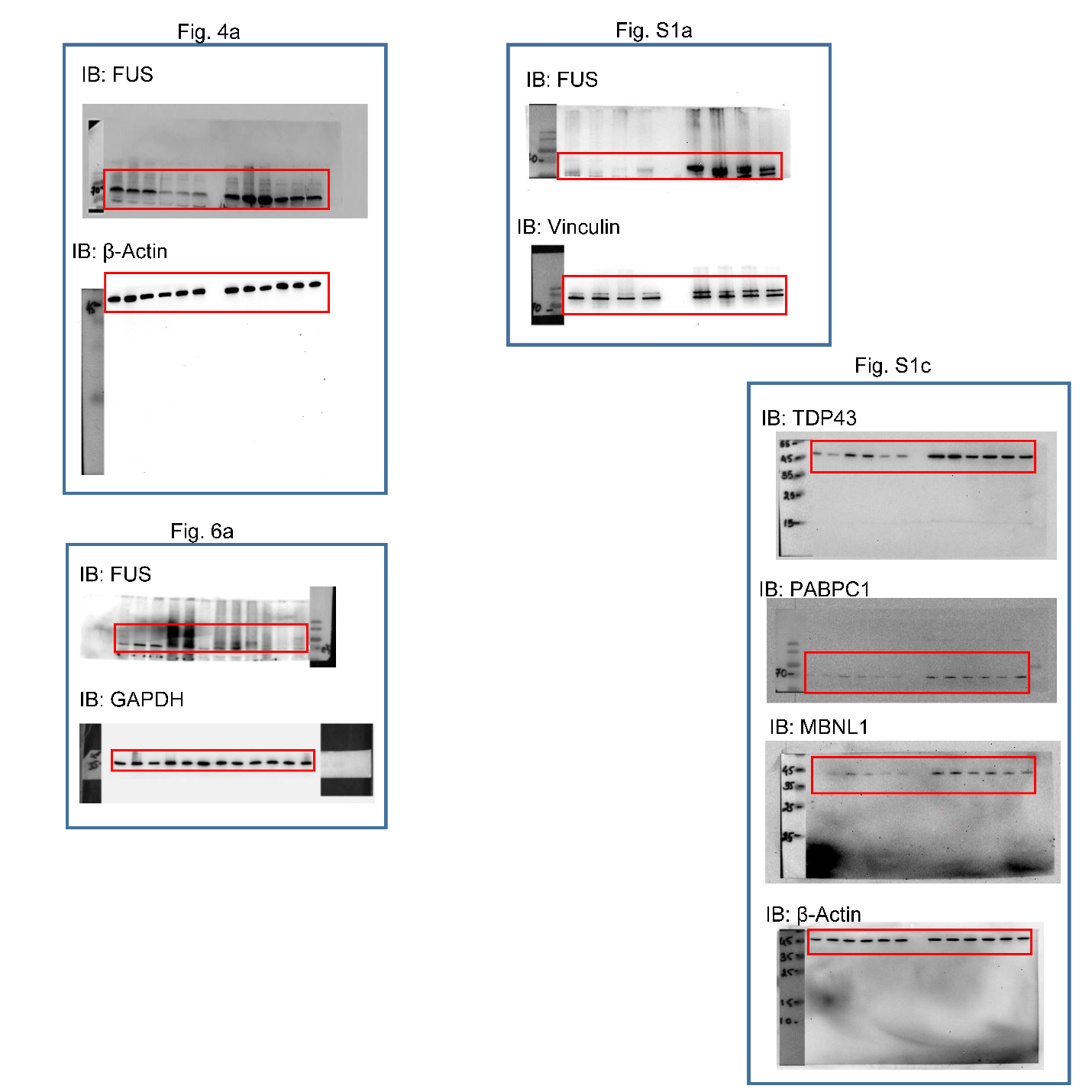


Appendix

Uncropped western blots of the indicated figure numbers shown in this study.
